# The Gender Life Satisfaction/Depression Paradox

**DOI:** 10.1007/s11205-021-02740-5

**Published:** 2021-09-19

**Authors:** Leonardo Becchetti, Gianluigi Conzo

**Affiliations:** Department of Economics and Finance, University of Rome Tor Vergata, Via Columbia 2, 00133 Rome, Italy

**Keywords:** Gender life satisfaction/depression paradox, Subjective wellbeing, I30, I31

## Abstract

According to the gender life satisfaction/depression paradox women are significantly more likely to report higher levels of life satisfaction than men after controlling for all relevant socio-demographic factors, but also significantly more likely to declare they are depressed. We find that the paradox holds in the cross-country sample of the European Social Survey and is stable across age, education, self-assessed health, macroregion and survey round splits. We find support for the affect intensity rationale showing that women are relatively more affected in their satisfaction about life by the good or bad events or achievements occurring during their existence and less resilient (less likely to revert to their standard levels of happiness after a shock). We as well discuss biological, genetic, cultural, personality rationales advocated in the literature that can explain our findings.

## Introduction

Empirical findings in the subjective wellbeing literature highlight a gender paradox. Women are more likely to fall into depression and, at the same time, declare higher levels of life satisfaction. On the first point Kessler et al. (1993) show in their national comorbidity survey that lifetime prevalence of a major depressive disorder in women (21.3%) is almost twice as large as in men (12.7%). Weissman et al. (1996) obtain similar findings in 10 different countries (United States, Canada, Puerto Rico, France, West Germany, Italy, Lebanon, Taiwan, Korea, and New Zealand). On the second point Nolen-Hoeksema and Rusting (1999) in their comprehensive review observe that women report more life satisfaction and more intense positive emotions than men. More recently, Matteucci and Lima (2016) test for the gender gap in life satisfaction in 85 countries in the 1981–2009 period finding a prevalence for higher female life satisfaction (71 percent of cases in 136 different country-year estimates). Inglehart (2002) analyses the dynamics of the gender life satisfaction gap and shows that it is growing in developing countries while falling in developed countries.

The evidence reported above indicates that what we define as the women life satisfaction/depression paradox is composed of two findings (prevalence of women depression and gender life satisfaction gap with women being relatively more satisfied) that have been studied separately.

The literature has provided several rationales to explain the different aspects of the paradox. A first rationale for women prevalence of depression is biological and related to changes in the endocrine control of the reproductive system in different stages (menstrual cycle, postpartum depression, menopause) of their life (Rudolf Noble 2005). The literature as well argues that the phenomenon cannot be explained only by these changes so that other psychosocial and/or metabolic factors enhance the original effect. This is consistent with evidence on the association of depression with several social controls.

A personality based interpretation is the affect intensity rationale arguing that women experience both positive and negative emotions with higher intensity than men (Diener et al. 1985). Further support for this rationale is found by Fujita et al. (1991) observing that women experience more negative affect than men but the same level of life satisfaction. Their experiment on a small sample of college students finds the rationale for the paradox in the different affect intensity since women report more intense positive emotions together with higher negative affect. Bryant et al. (1996) observe that women react more to negative than positive stimuli, while Brody and Hall (2008) show that women express stronger emotionality and argue that this is the case because they are educated to do so. Archer et al. (2007) study coping behavior and affect using the Positive and Negative Affect Scales (PANAS). Their empirical findings on the relationship among personality characteristics, gender and affect report a higher level of responsibility and vigor among female participants, together with more psychological stress and greater emotional coping vis-à-vis male participants. In the same direction, higher women caring responsibilities contribute to increase quality of relational life and therefore life sense and satisfaction but also dependence of one’s own satisfaction from the behaviour of other people, thereby exposing them more to relational shocks and affect related negative emotions. Carstensen et al. (2020) with their experiment on exposure to prolonged, unescapable stress conditions such as those of the COVID-19 pandemic find that female gender accounts for significantly more negative reactions and significantly less positive reactions vis-à-vis male gender after controlling for all relevant concurring factors. These findings are consistent with the affect intensity rationale of the gender life satisfaction/depression paradox.

Whenever a difference between sexes arises the debate on whether it is genetic or cultural also comes in parallel. A genetic rationale for the gender life satisfaction gap is advocated by Chen et al. (2013). The authors run their research on a US-representative cohort of New York residents and find that the MAOA-L (the low expression allele of the MAOA gene) predicts higher life satisfaction. The gene is present in females while not in males where the hormone testosterone plays an antagonist effect. On the other side the cultural interpretation theories of the socialization imprint on gender differences (e.g., Chodorow 1978; Gilligan 1982) argue that boys are trained to be competitive and to control their emotions, while girls to be more emphatic and caring (e.g., Keller and Scharff-Goldhaber 1987; Merchant 1979) according to a traditional distinction of roles.

Further evidence on gender differences that may ultimately affect the stated paradox comes from experimental findings in behavioral economics. In their survey on these studies Croson and Gneezy (2009) argue that the three main differences between sexes imply that women are more inequity averse, risk averse and competition averse, while the authors do not observe significant differences in trust, trustworthiness or social capital. Future research in behavioral economics should investigate whether evidence on gender differences in the experimental literature can be somewhat related to the gender happiness paradox, to its affect intensity rationale and to the socialization imprint.

To sum up, evidence coming from studies of gender effects on depression and subjective wellbeing highlights the existence of the paradox. The rationales provided are biological, cultural, related to personality traits and genetic.

We aim to contribute to this literature in several respects. First, we put together the two elements of the paradox empirically in the same paper and therefore we observe the two elements in a large cross-country sample in the same period. Second, in the light of the dynamics on the gender life satisfaction gap observed by Inglehart (2002) and Matteucci and Lima (2016), we want to see whether the paradox is robust when considering different geographical areas, sample periods, education, age and self-reported health levels. Third, we test whether the affect intensity hypothesis may be a good rationale for our findings by checking whether there is a significantly different gendered reaction to positive and negative events and achievements during life. Fourth, we go further in depth on this point with a specific focus testing whether there is a gender difference in resilience that is, in the positive reaction to negative shocks. Fifth, we evaluate whether the paradox is weaker or disappears in subsamples of women who are more educated, younger or living in Scandinavian countries where the cultural rationale of the paradox (ie. its origin depending on culturally based gender job inequality and/or domestic task overload on females) should matter less. As is well known female labor market participation in Scandinavian countries is well above OECD average (around 70% against 50.2%) and the female/male unpaid work hour ratio is on average around 2 in OECD countries against 1.6 in Scandinavian countries. To interpret the effect of these two factors on the life satisfaction/depression paradox we refer to some contributions of the recent literature. Nordenmark (2017) identifies different gender regimes across European regions and characterizes the Scandinavian as based on higher female labour market participation, economic independence, gender equality and more equal division of housework. The following empirical analysis shows that women life (but not family) satisfaction is generally higher under the Scandinavian regime. Álvarez and Miles-Touya (2016) find that being responsible for most of the housework reduces life satisfaction for full-time female workers. However the same findings show that women can still have high levels of life satisfaction with most of the housework when having part time jobs. This is more likely to occur when they have more conservative values, caring responsibilities and lower education. Başlevent and Kirmanoğlu (2017) work on European Social Survey data and show that working women have higher wellbeing than housewives in countries with a smaller gender gap measured with the GGGI (World Economic Forum’s Global Gender Gap Index), that is in countries with more gender equality in public domain positions. Overall this research shows an articulate picture by indicating that not always and in all cases higher labor market participation and reduced housework contribute to increase female life satisfaction. This is more likely to occur when important co-factors are at work such as gender equality in the job market (and high education or self-reported female non conservative values). This is however the case of the Scandinavian society where higher female labor market participation, more equal housework gender balance and more gender equality in the job market jointly apply. Based on our work and on the previous research we may therefore infer the existence of two equilibria equally contributing to female happiness. The first is high education and job market participation, gender balance in the job market and housework time. The second is caring responsibilities, conservative values, lack of gender balance in the job market and housework time. Even though the literature shows that women are happy also under this second equilibrium the gender life satisfaction/depression paradox is more likely to occur here where the negative side of external factors not under women control (increasing responsibility and gender imbalance in job market and household task) is more likely to produce mixed outcomes of life satisfaction (when external factors are under control) and depression (when they end up being out of control). Based on this evidence we test the hypothesis that the gender happiness gap is different in Scandinavian countries.

Our empirical findings show that the gender paradox is confirmed and relevant in magnitude since women have an almost 0.2 percent higher probability to declare the highest (10) life satisfaction level and an around 4 percent higher probability to declare a level of happiness higher than 7. At the same time they have a 2 percent higher probability to declare that they have been depressed most of times or all time in the last week.

Our results as well support the hypothesis that the gender paradox is extremely stable across geographical areas, survey rounds, education, age and self-assessed health splits. We as well test whether the affect intensity hypothesis can explain the paradox and find that it is the case. The impact of standard (positive and negative) drivers of life satisfaction (related to events or achievements) is significantly higher for women than for men since gender interacted variables are all significant after controlling for the standard variable effect. In addition to it, we find that women are less resilient that is, they declare to take significantly more time to revert to their previous wellbeing levels after a negative shock. This gender resilience effect contributes to explain the negative side of the affect intensity rationale.

Results on the Scandinavian/non Scandinavian difference find higher gender life satisfaction difference in Scandinavian countries (even though high standard errors in the two subsample estimates tell us that the difference is not statistically significant), while the gender depression difference remains the same. This obviously does not imply that the paradox could not be explained by cultural and socioeconomic factors, while just that the observable cultural and socioeconomic factors considered in this sample split do not fully account for it.

## The Database

Data for our empirical analysis come from the European Social Survey (ESS). We take into consideration the fourth, fifth, sixth, seventh and eight waves of ESS implemented in 2008, 2010, 2012, 2014 and 2016 respectively. The ESS collects information on social and political preferences and beliefs and socio-demographic variables of a large sample of respondents of Europeans aged 15 and over. Waves 4-7 include (for at least one wave) representative samples from the following 35 countries: Albania, Austria, Germany, Sweden, Netherlands, Norway, Spain, Finland, Italy, France, Denmark, Greece, Switzerland, Belgium, Iceland, Israel, Bulgaria, Cyprus, United Kingdom, Czech Republic, Poland, Ireland, Ukraine, Turkey, Kosovo Hungary, Slovakia, Portugal, Slovenia, Estonia, Romania, Russian Federation, Lithuania, Latvia and Croatia. Note however that the depression variable is present only in waves 3, 6 and 7 (and therefore in 31 countries for at least one wave), while the life satisfaction variable is present in waves 3, 4, 5, 6 and 7 (and therefore in 35 countries for at least one wave). The countries dropped for lack of data on depression are Romania, Turkey, Croatia and Greece. To avoid that our findings can be driven by differences in the two samples we will test whether the paradox holds in specific single waves (see Table 5 that follows). The life satisfaction/depresssion paradox can be therefore measured only for 31 countries who have data for both life satisfaction and depression.

## Empirical Findings

The variable legend is in Table 1 while descriptive findings of the variables used in the econometric analysis are presented in Table 2. The sample is well balanced, concerning gender, since slightly less than half of it is composed by males (46.25 percent). The average number of members in the household is 2.71, and around 20.3 percent of the respondents find it difficult to live with the present income, while 27.6 percent of the sample have never been married.

**Table 1:** Variable legend

Variable	Description
Dependent variables	
Life satisfaction	Self-assessed life satisfaction scores (0–10)
Depression	$\hbox {Dummy variable}=1$ if the respondent is depressed and 0 otherwise.
Socio-demographic and other variables	
Age class	0/1dummies for the following age groups: Age 0–19; Age 20–29; Age 30–39; Age 40–49; Age 50–59; Age 60–69; Age 70–79; Age 80–89; Age 90+.
Education status	ISCED (International Standard Classification of Education) levels: Zero level of education meaning no education or unfinished first level of education. First level (primary education or first stage basic education), second level (lower secondary or second stage of basic education), third level (upper secondary education), fourth level (post-secondary non tertiary education), fifth level (first stage of tertiary education), sixth level (second stage of tertiary education).
Female	$\hbox {Dummy variable} = 1$ if the respondent’s gender is female and 0 otherwise.
Employment status	Employment status categorical variable: $1=\hbox {Paid worker}$, $2=\hbox {Student}$, $3=\hbox {Unemployed}$ (active), $4=\hbox {Unemployed}$ (not active), $5=\hbox {Retired}$.
Income	Yearly household income after taxes and social insurance contributions.
Marital status	Marital status categorical variable: $1=\hbox {Married}$, $2=\hbox {Registered Partner}$; $3=\hbox {Divorced}$ $4=\hbox {Separated}$; $5=\hbox {Widowed}$
Household Size	Number of people leaving regularly as member of the household
Self health	Subjective general health categorical variable: $1=\text {Very good health}$, $2=\hbox {Good health}$, $3=\hbox {Fair health}$, $4=\hbox {bad Health}$, $4=\hbox {Very bad health}$.
Social meeting	Categorical variable that measures how often socially meet with friends, relatives or colleagues: $1=\hbox {Never}$, $2=\hbox {Less than once a month}$, $3=\hbox {Once a month}$, $4=\hbox {Several times a month}$, $5=\hbox {Once a week}$, $6=\hbox {Several times a week}$, $7=\hbox {Every day}$.
Placement on the left right scale	Categorical variable that indicates political preferences based on a 0–10 scale. The 0 is associated with the extreme left political preference, while 10 is associated with the extreme right political preference.
Resilience	Categorical variable that indicates if things go wrong, the time to get back to the previous situation. $1=\hbox {Very long time}$, $2=\hbox {Long time}$, $3=\hbox {Neither long nor short}$, $4=\hbox {Short time}$, $5=\hbox {Very short time}$
Proxy for the Wealth/Feeling about income	Categorical variable that indicates the feeling about the income nowadays, in this case is used as proxy for the Wealth. $1=\hbox {Living comfortably on present income}$, $2=\hbox {Copying on present income}$, $3=\hbox {Difficult on present income}$, $4=\hbox {Very difficult on present income}$.
Wave	2008 wave, 2010 wave, 2012 wave, 2014 wave, 2016 wave.
Country	The countries where the surveys were realized: Albania, Austria, Germany, Sweden, Netherlands, Norway, Spain, Finland, Italy, France, Denmark, Greece, Switzerland, Belgium, Iceland, Israel, Bulgaria, Cyprus, United Kingdom, Czech Republic, Poland, Ireland, Ukraine, Turkey, Kosovo, Hungary, Slovakia, Portugal, Slovenia, Estonia, Romania, Russian Federation, Lithuania, Latvia and Croatia

**Table 2:** Descriptive statistics

Depression variable	Obs	Density	Variable	Obs	Density
*Depression*	94.269	0.076	*If things go wrong, time to get back to normal (Proxy for resilience)*	53.946	
*Life Satisfation*	253.117		Very long time		0.049
0		0.008	Long time		0.211
1		0.007	Neither long nor short		0.229
3		0.030	Short time		0.404
4		0.038	Very short time		0.104
5		0.109			
6		0.090	*Social meeting*	253.114	
7		0.179	Never		0.021
8		0.263	Less than once a month		0.091
9		0.156	Once a month		0.099
10		0.100	Several times a month		0.196
			Once a week		0.176
*Household’s total net income*	197.985		Several times a week		0.263
1		0.102	Every day		0.151
2		0.114			
3		0.114	*Self health*	254.563	
4		0.112	Very good		0.235
5		0.108	Good		0.407
6		0.102	Fair		0.272
7		0.099	Bad		0.074
8		0.093	Very bad		0.015
9		0.076			
10		0.075	*Feeling about Household’s income nowadays*	191.608	
			Living comfortably on present income		0.263
Female	254.872	0.539	Copying on present income		0.443
			Difficult on present income		0.203
*Age class*	254.971		Very difficult on present income		0.089
0–19		0.056			
20–29		0.138	*Placement on left right scale*	217.664	
30–39		0.157	0		0.036
40–49		0.167	1		0.024
50–59		0.169	2		0.546
60–69		0.156	3		0.096
70–79		0.105	4		0.097
80–89		0.041	5		0.328
90+		0.006	6		0.098
			7		0.106
			8		0.084
*Country*	254.971		9		0.029
Albania		0.004	10		0.042
Austria		0.032			
Belgium		0.034	*Education status*	254.971	
Bulgaria		0.027	No or unfinished		0.089
Switzerland		0.030	Primary		0.097
Cyprus		0.013	Lower Secondary		0.158
Czech Republic		0.042	Upper Secondary		0.148
Germany		0.057	Post-Secondary, non Tertiary		0.191
Denmark		0.024	First level Tertiary		0.155
Estonia		0.038	Second level Tertiary		0.087
Spain		0.040	Upper Tertiary		0.108
Finland		0.040			
France		0.038	*Marital Status*	254.971	
United Kingdom		0.044	Married		0.136
Greece		0.018	Registered partner		0.008
Croatia		0.012	Separated		0.004
Hungary		0.033	Never married		0.276
Ireland		0.047	Divorced		0.088
Israel		0.048	Widowed		0.096
Iceland		0.006			
Italy		0.014	*Household size*	254.619	2.711
Lithuania		0.039			
Latvia		0.007	*Employment Status*	254,971	
Netherlands		0.035	Paid Work		0.507
Norway		0.030	Student		0.097
Poland		0.033	Unemployed, active		0.049
Portugal		0.036	Unempolyed, not active		0.022
Romania		0.008	Retired		0.263
Russian Federation	0.039				
Sweden		0.033	*ESS Round*	254.971	
Slovenia		0.025	4		0.239
Slovakia		0.021	5		0.214
Turkey		0.009	6		0.214
Ukraine		0.023	7		0.157
Kosovo		0.005	8		0.174

**Fig. 1:**
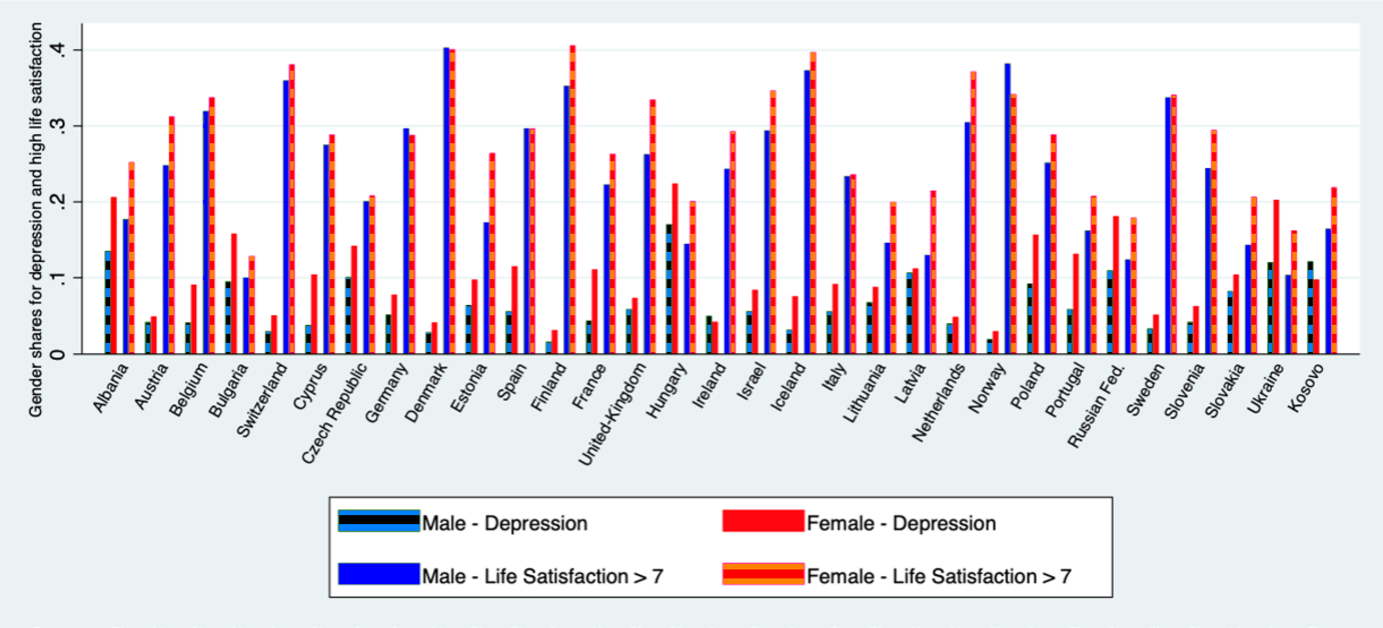
Depression and High Life Satisfaction across different countries. (average values across sample period)

Figure 1 provides descriptive evidence on the two parts of the gender paradox for each country in the sample and shows that the paradox (women reporting high life satisfaction for a higher share while, at the same time, depression for a higher share) applies to all of the 31 countries with the exception of Germany, Norway and Denmark (slightly higher life satisfaction for men), and Ireland and Kosovo (higher share of depression among men). Figure 1 also shows that women in Finland are the gender group with the highest level of life satisfaction in our cross-country sample, while men in Ukraine and Bulgaria the group with the lowest level.

In order to test whether descriptive evidence on the gender paradox is confirmed after controlling for concurring factors affecting our dependent variables we estimate the following econometric specification:

$$\begin{aligned}&{Subjective Wellbeing}_{i,t} \\&\quad =\alpha _{0} + \alpha _{1}{Female}_{i,t} +\sum _{g} \beta _{g} {D Age Class}_{i,t} +\sum _{j} \gamma _{j} {D Income Deciles}_{i,t} \\&\qquad + \sum _{k} \delta _{k} {D Employment Status}_{i,t} +\alpha _{2}{Household Size}_{i,t} \\&\qquad + \sum _{k} \zeta _{v} {D Marital Status}_{i,t} +\sum _{l} \theta _{l} {D Education Status}_{i,t} \\&\qquad + \sum _{m} \iota _{m} {D Self Health}_{i,t} +\sum _{o} \lambda _{o} {D Left Right Scale}_{i,t} \\&\qquad + \sum _{p} \rho _{p} {D Social Meeting}_{i,t} +\sum _{q} \phi _{q} {D Feeling On Income}_{i,t} \\&\qquad + \sum _{r} \chi _{r} {D Country}_{i,t} +\sum _{s} \psi _{s} {D Wave}_{i,t} + \epsilon _{i,t} \end{aligned}$$

where the dependent variable (*SubjectiveWellbeing*) is a 0/1 dummy taking unit value if the respondent answers that she/he has been depressed most of times or all time in the last week in the depression estimate (Table 3, column 1) and a discrete qualitative variable measuring life satisfaction levels in the standard 0–10 scale in the life satisfaction estimate (Table 3, column 2). Controls include a (0/1) dummy for female gender, (five year) age classes, dummies for income deciles and a variable measuring household size. The estimate includes dummies for the employment status of the respondent (student, unemployed inactive, unemployed active and retired) with paid workers being the omitted benchmark. Marital status dummies include the following conditions (in a civil partnership, formerly in civil partnership, now dissolved, formerly in civil partnership, partner died, separated (still legally married), separated (still in a civil partnership), divorced, widowed, never married and never in civil partnership) with the married status omitted benchmark. The estimate also includes dummies for education based on the standard ISCED2 classification (less than lower secondary, lower secondary, lower tier upper secondary, upper tier upper secondary, advanced vocational, sub-degree, lower tertiary education, higher tertiary education). The upper tertiary class in the ISCED^1^ classification is used as omitted benchmark. Self-assessed health is measured with four dummies (good, fair, bad, very bad) with very good being the omitted benchmark. Controls about the political preferences and the frequency of social meeting are also added to the estimate. More specifically, for the political preferences, the estimate includes dummies for placement on a 0–10 left-right political scale, with the extreme left (0 class) being the omitted benchmark. The control for social interactions introduces dummies for the frequency of social meetings (Less than once a month, Once a month, Several times a month, Once a week, Several times a week, Every day) with “never” being the omitted benchmark. Feeling for present income is measured by three dummies (coping on present income, difficult, very difficult) with living comfortably being the omitted benchmark).

**Table 3:** The gender happiness paradox (determinants of life satisfaction and depression)

Variables	Probit	Ordered Probit	OLS
	(1)	(2)	(3)
	Depression	Life satisfaction	Life satisfaction
*Female*	0.178***	0.116***	0.175***
	(0.030)	(0.011)	(0.017)
*Age class*			
0–19	0.140**	0.135***	0.214***
	(0.058)	(0.024)	(0.041)
20–29	0.071**	0.133***	0.211***
	(0.029)	(0.015)	(0.024)
30–39	0.030	0.078***	0.116***
	(0.030)	(0.010)	(0.017)
50–59	−0.071***	0.031***	0.051***
	(0.019)	(0.012)	(0.018)
60–69	−0.177***	0.123***	0.186***
	(0.032)	(0.024)	(0.036)
70–79	−0.242***	0.197***	0.301***
	(0.042)	(0.028)	(0.039)
80–89	−0.244***	0.253***	0.390***
	(0.041)	(0.038)	(0.057)
90+	−0.220**	0.254***	0.388***
	(0.092)	(0.044)	(0.066)
*Employment status*			
Student	−0.031	0.040***	0.083***
	(0.032)	(0.013)	(0.021)
Unemployed, active	0.138***	−0.175***	−0.321***
	(0.033)	(0.021)	(0.039)
Unemployed, not active	0.174***	−0.141***	−0.260***
	(0.048)	(0.022)	(0.038)
Retired	−0.033	0.061***	0.091***
	(0.031)	(0.012)	(0.019)
*Household’s income*			
2	−0.007	0.016	0.062**
	(0.038)	(0.014)	(0.027)
3	−0.034	0.026	0.087***
	(0.037)	(0.017)	(0.031)
4	−0.038	0.019	0.083**
	(0.030)	(0.018)	(0.032)
5	−0.057	0.032	0.107***
	(0.037)	(0.020)	(0.036)
6	−0.071*	0.044**	0.135***
	(0.039)	(0.019)	(0.033)
7	−0.062	0.066***	0.168***
	(0.047)	(0.022)	(0.037)
8	−0.085**	0.061***	0.166***
	(0.040)	(0.022)	(0.038)
9	−0.087*	0.075***	0.190***
	(0.047)	(0.025)	(0.040)
10	−0.200***	0.086***	0.188***
	(0.064)	(0.024)	(0.040)
*Household size*	−0.013	0.044***	0.066***
	(0.009)	(0.005)	(0.007)
*Education status*			
No or unfinished	0.039	0.084*	0.102
	(0.055)	(0.046)	(0.070)
Primary	0.203***	0.080***	0.061
	(0.037)	(0.028)	(0.039)
Lower Secondary	0.154***	0.009	−0.038
	(0.035)	(0.028)	(0.042)
Upper Secondary	0.044	0.019	−0.006
	(0.028)	(0.020)	(0.029)
Post-Secondary	0.074**	−0.003	−0.030
	(0.030)	(0.018)	(0.027)
First Level Tertiary	0.029	0.029*	0.023
	(0.029)	(0.016)	(0.023)
Second Level Tertiary	0.026	0.003	−0.002
	(0.040)	(0.011)	(0.018)
*Marital status*			
Registered partner	0.095	−0.165***	−0.265***
	(0.067)	(0.037)	(0.064)
Separated	0.314***	−0.464***	−0.751***
	(0.067)	(0.032)	(0.045)
Divorced	0.110***	−0.253***	−0.404***
	(0.025)	(0.015)	(0.028)
Widowed	0.175***	−0.315***	−0.500***
	(0.028)	(0.029)	(0.044)
Never Married	0.037*	−0.271***	−0.399***
	(0.019)	(0.017)	(0.027)
*Self health*			
Good	0.206***	−0.318***	−0.426***
	(0.030)	(0.017)	(0.027)
Fair	0.579***	−0.601***	−0.890***
	(0.033)	(0.026)	(0.040)
Bad	1.101***	−0.922***	−1.514***
	(0.043)	(0.036)	(0.056)
Very Bad	1.584***	−1.255***	−2.251***
	(0.070)	(0.041)	(0.072)
*Social meeting*			
Less than once a month	−0.291***	0.199***	0.467***
	(0.056)	(0.040)	(0.073)
Once a month	−0.414***	0.358***	0.776***
	(0.061)	(0.041)	(0.074)
Several times a month	−0.568***	0.459***	0.955***
	(0.050)	(0.039)	(0.071)
Once a week	−0.501***	0.491***	1.001***
	(0.052)	(0.041)	(0.073)
Several times a week	−0.572***	0.610***	1.178***
	(0.049)	(0.040)	(0.072)
Every day	−0.525***	0.738***	1.336***
	(0.048)	(0.038)	(0.071)
*Placement on left right scale*			
1	−0.098**	−0.102***	−0.111***
	(0.039)	(0.021)	(0.034)
2	−0.077*	−0.155***	−0.169***
	(0.043)	(0.024)	(0.040)
3	−0.077**	−0.174***	−0.177***
	(0.033)	(0.027)	(0.043)
4	−0.049	−0.190***	−0.192***
	(0.041)	(0.023)	(0.036)
5	−0.116***	−0.078***	−0.040
	(0.029)	(0.027)	(0.045)
6	−0.099***	−0.128***	−0.083
	(0.036)	(0.031)	(0.049)
7	−0.136***	−0.107***	−0.052
	(0.038)	(0.029)	(0.047)
8	−0.049	0.015	0.109*
	(0.034)	(0.032)	(0.054)
9	−0.116**	0.094***	0.230***
	(0.057)	(0.030)	(0.051)
10	−0.049	0.236***	0.361***
	(0.051)	(0.043)	(0.067)
*Feeling about Household’s income nowadays*			
Copying on present income	0.097***	−0.238***	−0.312***
	(0.022)	(0.015)	(0.026)
Difficult on present income	0.334***	−0.519***	−0.801***
	(0.031)	(0.030)	(0.052)
Very difficult on present income	0.627***	−0.804***	−1.405***
	(0.040)	(0.037)	(0.073)
*Wave dummies*	Yes	Yes	Yes
*Country dummies*	Yes	Yes	Yes
*Constant*	−1.314***		6.278***
	(0.062)		(0.100)
*Observations*	92,582	197,882	197,882
*Pseudo R-Squared*	0.178	0.0816	
*R-Squared*			0.297
Country effects			
*Country*			
Austria	−0.327***	0.233***	0.442***
	(0.031)	(0.029)	(0.049)
Belgium	−0.119***	0.390***	0.768***
	(0.030)	(0.020)	(0.034)
Bulgaria	−0.297***	−0.394***	−0.689***
	(0.023)	(0.024)	(0.034)
Switzerland	−0.218***	0.530***	0.916***
	(0.034)	(0.022)	(0.037)
Cyprus	−0.252***	0.331***	0.666***
	(0.022)	(0.024)	(0.041)
Czech Republic	0.177***	0.011	0.137***
	(0.027)	(0.019)	(0.030)
Germany	−0.181***	0.381***	0.697***
	(0.031)	(0.018)	(0.031)
Denmark	−0.317***	0.674***	1.098***
	(0.031)	(0.026)	(0.041)
Estonia	−0.265***	0.230***	0.466***
	(0.021)	(0.018)	(0.030)
Spain	−0.173***	0.433***	0.827***
	(0.027)	(0.017)	(0.029)
Finland	−0.646***	0.630***	1.086***
	(0.027)	(0.030)	(0.049)
France	−0.079***	0.097***	0.305***
	(0.029)	(0.018)	(0.032)
United Kingdom	−0.198***	0.311***	0.599***
	(0.028)	(0.028)	(0.047)
Greece		−0.236***	−0.262***
		(0.034)	(0.050)
Croatia		−0.073***	0.004
		(0.022)	(0.037)
Hungary	0.039	0.013	0.072**
	(0.024)	(0.018)	(0.027)
Ireland	−0.317***	0.174***	0.378***
	(0.026)	(0.025)	(0.043)
Israel	−0.148***	0.298***	0.582***
	(0.020)	(0.023)	(0.041)
Iceland	−0.192***	0.529***	0.911***
	(0.018)	(0.023)	(0.037)
Italy	−0.239***	0.117***	0.350***
	(0.016)	(0.016)	(0.028)
Lithuania	−0.336***	−0.041*	−0.000
	(0.027)	(0.023)	(0.036)
Latvia	−0.214***	0.015	0.118**
	(0.046)	(0.031)	(0.048)
Netherlands	−0.348***	0.390***	0.789***
	(0.032)	(0.020)	(0.034)
Norway	−0.503***	0.482***	0.845***
	(0.029)	(0.024)	(0.040)
Poland	0.005	0.351***	0.630***
	(0.029)	(0.017)	(0.029)
Portugal	−0.258***	−0.017	0.106***
	(0.025)	(0.021)	(0.033)
Romania		−0.042	0.045
		(0.029)	(0.044)
Russian Federatio	−0.129***	0.011	0.052
	(0.032)	(0.021)	(0.031)
Sweden	−0.216***	0.338***	0.652***
	(0.030)	(0.030)	(0.052)
Slovenia	−0.463***	0.228***	0.466***
	(0.026)	(0.017)	(0.031)
Slovakia	−0.173***	0.054***	0.222***
	(0.030)	(0.017)	(0.028)
Turkey		−0.626***	−1.140***
			(0.070)
Ukraine	−0.107***	−0.110***	−0.177***
	(0.024)	(0.022)	(0.032)
Kosovo	−0.054***	−0.227***	−0.277***
	(0.016)	(0.010)	(0.015)
*Observations*	92,582	197,882	197,882
*Pseudo R-Squared*	0.178	0.0816	
*R-Squared*			0.297

Omitted benchmarks: age class between 40 and 49; paid workers class of the employment relation; first (lowest income) class of the household’s net income; “Upper Tertiary” class for education status; “Married” class for marital status; “Very Good” class of self-assessed health; “Never” class of social meeting; the 0 (extreme left) class of placement in the political opinion left-right scale; “Living Comfortably on present income” in the Feeling about Household’s income question, Albania for country dummies. Dependent variable is “depression” in (1), while life satisfaction in (2) and (3). Estimates in (1) and (2) are obtained using the “probit” (and ordered-probit) regression, while in (3) using the “OLS” regression. Clustered (for country) standard errors in parentheses.

*** *p*<0.01, ** *p*<0.05, *p<0.1

The estimate finally includes dummies for each country of origin (Austria, Germany, Sweden, Netherlands, Norway, Spain, Finland, Italy, France, Denmark, Greece, Switzerland, Belgium, Iceland, Israel, Bulgaria, Cyprus, United Kingdom, Czech Republic, Poland, Ireland, Ukraine, Turkey, Kosovo Hungary, Slovakia, Portugal, Slovenia, Estonia, Romania, Russian Federation, Lithuania, Latvia and Croatia), with Albania being the omitted benchmark. The depression equation is estimated with a probit specification, while the life satisfaction equation is estimated with an ordered probit specification that properly acknowledges the discrete qualitative nature of our dependent variable. We however compare our ordered probit life satisfaction findings with those from an OLS model that is usually alternatively estimated in several papers of the life satisfaction literature finding results not significantly different from those of the ordered probit model. All models are estimated with country-clustered standard errors.

## Discussion of Empirical Findings

In Table 3 we present estimates of variables associated to depression (column 1) and life satisfaction (column 2) The hypothesis of the gender life satisfaction/depression paradox is not rejected since the female gender is significant and positive in both estimates. Our findings tell that women are more likely to be depressed, but also more likely to be highly satisfied with their lives. Based on the findings from the estimated specification shown in Table 2 we want to evaluate the economic significance of the gender effect that is, the contribution of gender to the realisation of our dependent variable everything else being equal. We therefore calculate the marginal contribution of female gender and find that it raises by 0.2 percent the probability of declaring the highest level of life satisfaction (that becomes a 4 percent higher probability of declaring a level above 7) and by 2 percent the probability that our respondents declare they have been depressed most of times in the last week. Other controls have the standard sign and significance. Self-assessed health and frequency of social meetings are positively and significantly correlated to life satisfaction and negatively to depression. The same occurs for income satisfaction. The age effect is U-shaped when we consider life-satisfaction as dependent variable and descending when regressed on depression, with older respondents less likely to declare they are depressed. Unemployed respondents are significantly more likely to be depressed and less satisfied with their life. Education is statistically associated with the two dependent variables, especially through the depressing (and life satisfaction decreasing) effect of not going beyond primary education. Respondents with a registered partner are slightly less likely to declare high satisfaction vis-à-vis the omitted benchmark of married respondents, but however more satisfied with life and less depressed than separated, divorced and never married, consistently with previous literature. The effect of political orientation is less clear cut with respondents with moderate left or center orientation being less likely to be depressed or less likely to be highly satisfied than extreme left respondents.

Characteristics of our database allow us to test the stability of the paradox, a relevant issue given the literature findings on the dynamics and time variability of the gender life satisfaction effect (Inglehart 2002; Matteucci and Lima 2016). Our findings on this point show that the gender life satisfaction/depression paradox is robust as it persists when we split the sample based on age, education, self-assessed health and geographical areas (main findings in Tables 4 while full details of subsample estimates in the Appendix Tables 11 and 12). It remains robust as well when we estimate the model wave by wave and separately for different seasons of the year in order to check whether the life satisfaction/depression paradox might depend from seasonal effects (main findings in Tables 5 and 6 while full details of subsample estimates in the Appendix Tables 13 and 14). Table 5 shows that the difference in magnitudes of the female life satisfaction coefficients across waves are stronger than those of the depression coefficient and, in particular, those of waves 3 and 6 compared to wave 7. However the standard errors tell us that the confidence intervals of the two coefficients do overlap. We as well perform a test on the significance of the statistical difference of the female happiness coefficient in wave 7 by estimating a unique equation with a dummy interacted variable for the seventh wave. The coefficient is not significantly different.

**Table 4:** “Female” coefficients across different subsamples

	Age 0–59	Age 60–90+	Low Level Education	High Level Education	Not Good Self Health	Good Self Health	With Eastern countries	Without Eastern countries
Dependent Variable: *Depression*								
Female	0.184***	0.168***	0.204***	0.147***	0.175***	0.179***	0.141***	0.195***
	(0.033)	(0.036)	(0.034)	(0.034)	(0.038)	(0.033)	(0.029)	(0.042)
Dependent Variable: *Life satisfaction*								
Female	0.129***	0.092***	0.097***	0.132***	0.121***	0.109***	0.122***	0.114***
	(0.011)	(0.013)	(0.012)	(0.011)	(0.010)	(0.015)	(0.012)	(0.014)

**Table 5:** “Female” coefficients across different waves and regions

	Scandinavian Countries	No Scandinavian Countries	Waves 3-6-7	Wave 3	Wave 6	Wave 7
Dependent Variable: *Depression*						
Female	0.168***	0.179***	0.178***	0.181***	0.185***	0.173***
	(0.052)	(0.034)	(0.030)	(0.049)	(0.029)	(0.040)
Dependent Variable: *Life satisfaction*						
Female	0.156***	0.109***	0.115***	0.120***	0.133***	0.083***
	(0.034)	(0.011)	(0.010)	(0.016)	(0.015)	(0.014)

**Table 6:** “Female” coefficients across different seasons

	Summer	Autumn	Winter	Spring
Dependent Variable: *Depression*				
Female	0.151**	0.178***	0.180***	0.203***
	(0.065)	(0.033)	(0.039)	(0.067)
Dependent Variable: *Life satisfaction*				
Female	0.112***	0.114***	0.119***	0.113***
	(0.015)	(0.014)	(0.011)	(0.028)

The paradox remains unchanged when we re-estimate the model adding aggregate regional indicators (disposable income and unemployment rate), or use a specification with a time interaction between the female dummy and the year of the interview (Table 7).

**Table 7:** “Female” coefficients across different specifications

	Probit with Regional Indicators	Probit with Time Interaction
Dependent Variable: Depression		
Female	0.193***	0.203***
	(0.045)	(0.046)
Dependent Variable: Life Satisfaction		
Female	0.120***	0.129***
	(0.016)	(0.019)

In order to understand which factors explain the switch from the high (above 7) satisfaction to the depressed group among women we run a regression on the women sample only including those belonging to one of the two (highly satisfied/depressed) groups with depression being the dependent variable (Table 8). We find that the four main drivers explaining the difference between the two groups are that the depressed women are, in comparison to the highly satisfied, younger (with the exception of the 90+ age cohort), with lower education, poorer self-assessed health and in higher proportion in difficulty to cope with present income.

**Table 8:** The determinants of Depression (sample with very high life satisfaction or depressed females only)

Variables	Depression
*Age class*	
0–19	0.071
	(0.098)
20–29	0.221***
	(0.057)
30–39	0.080
	(0.052)
50–59	−0.039
	(0.030)
60–69	−0.070
	(0.058)
70–79	−0.192***
	(0.060)
80–89	−0.138*
	(0.071)
90+	0.123
	(0.111)
*Employment relation*	
Student	0.070
	(0.045)
Unemployed, active	0.029
	(0.054)
Unemployed, not active	0.095
	(0.089)
Retired	0.030
	(0.049)
*Household’s income*	
2	0.036
	(0.071)
3	0.076
	(0.057)
4	0.043
	(0.058)
5	0.012
	(0.060)
6	0.106
	(0.081)
7	0.093
	(0.077)
8	−0.018
	(0.082)
9	0.048
	(0.083)
10	−0.029
	(0.095)
*Household size*	0.016
	(0.015)
*Education status*	
No or unfinished	0.167*
	(0.096)
Primary	0.421***
	(0.085)
Lower Secondary	0.247***
	(0.067)
Upper Secondary	0.131**
	(0.063)
Post-Secondary	0.152**
	(0.061)
First Level Tertiary	0.117*
	(0.062)
Second Level Tertiary	0.116
	(0.082)
*Marital Status*	
Registered partner	−0.082
	(0.112)
Separated	−0.020
	(0.235)
Divorced	−0.105*
	(0.061)
Widowed	−0.027
	(0.059)
Never Married	−0.110**
	(0.049)
*Self health*	
Good	0.180***
	(0.046)
Fair	0.412***
	(0.038)
Bad	0.674***
	(0.076)
Very Bad	1.022***
	(0.108)
*Social meeting*	
Less than once a month	−0.121
	(0.111)
Once a month	−0.099
	(0.099)
Several times a month	−0.158*
	(0.083)
Once a week	−0.091
	(0.092)
Several times a week	−0.128
	(0.082)
Every day	−0.057
	(0.085)
*Placement on left right scale*	
1	−0.078
	(0.102)
2	−0.137*
	(0.075)
3	−0.126
	(0.079)
4	−0.077
	(0.081)
5	−0.115*
	(0.064)
6	−0.065
	(0.082)
7	−0.151***
	(0.056)
8	−0.013
	(0.072)
9	0.024
	(0.104)
10	0.079
	(0.071)
*Feeling about Household’s income nowadays*	
Copying on present income	−0.006
	(0.041)
Difficult on present income	0.039
	(0.054)
Very difficult on present income	0.155**
	(0.075)
*Waves*	
6	−0.042
	(0.055)
7	−0.115**
	(0.045)
*Country dummies*	Yes
*Constant*	−1.999***
	(0.149)
*Observations*	44,856
*Pseudo R-Squared*	0.0675

Sample survival indicates the marginal effects of the covariates on the survival across waves. Standard errors clustered at country level in parentheses.

*** $$p<0.01$$, ** $$p<0.05$$, * $$p<0.1$$

### Testing Rationales for the Gender Happiness Paradox

**Fig. 2:**
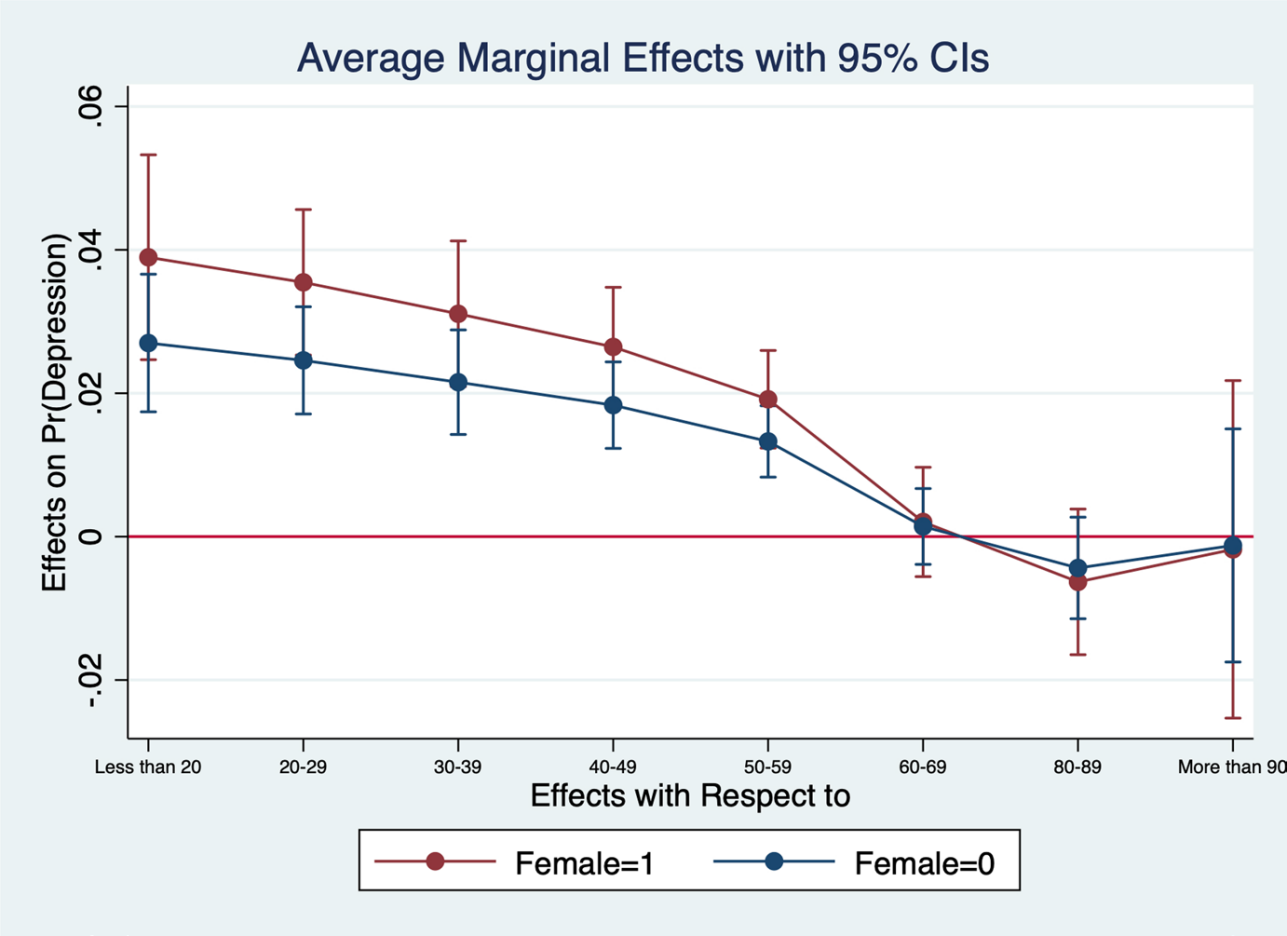
Gendered marginal effects: age classes. Table 3, Column (1) estimate. Average Marginal effect of age class on depression. The age class between 70 and 79 is the omitted benchmark

**Fig. 3:**
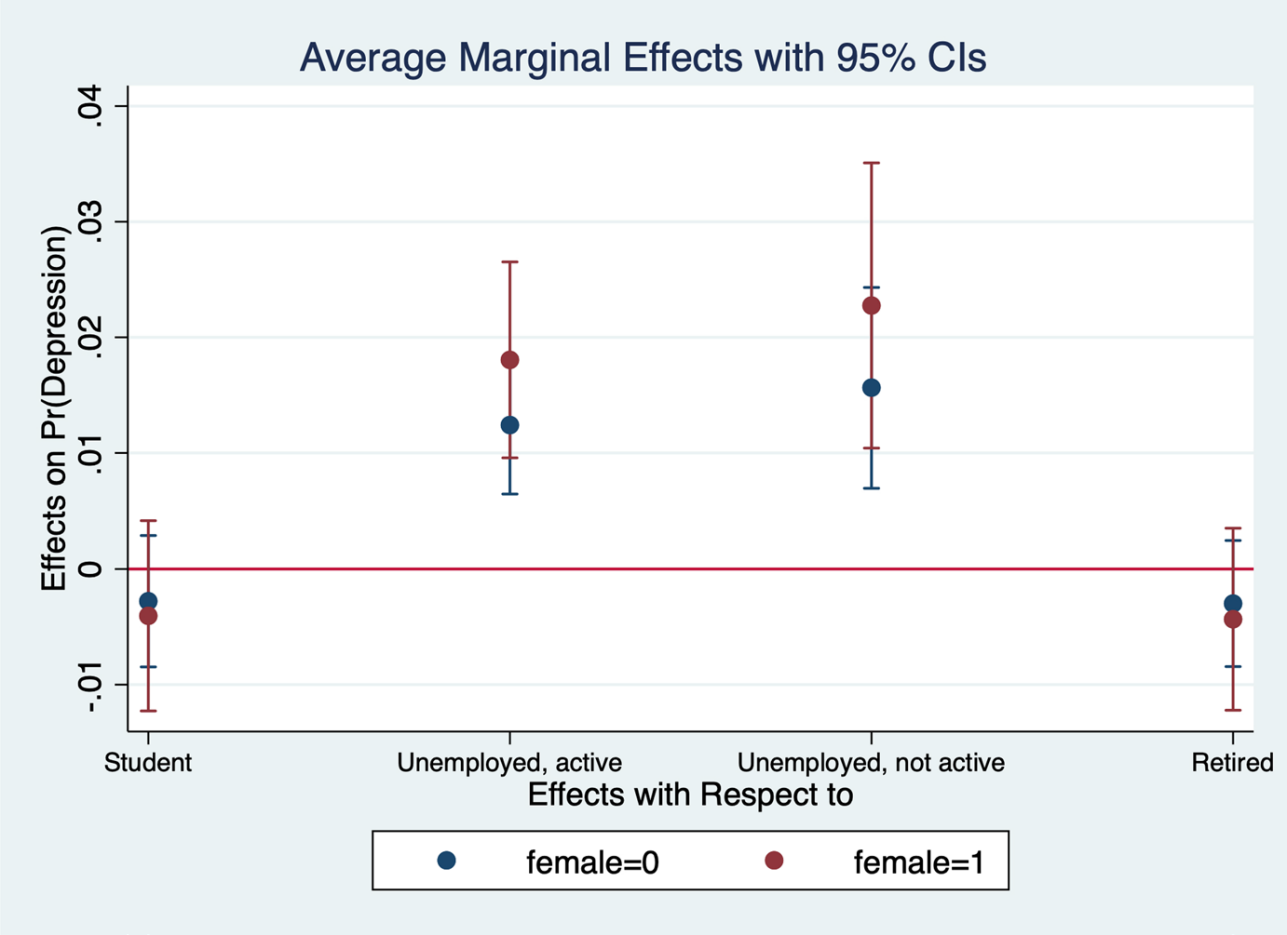
Gendered marginal effects: employment status. Table 3, Column (1) estimate. Average Marginal effect of employment status on depression. The “Paid Work class” is the omitted benchmark

**Fig. 4:**
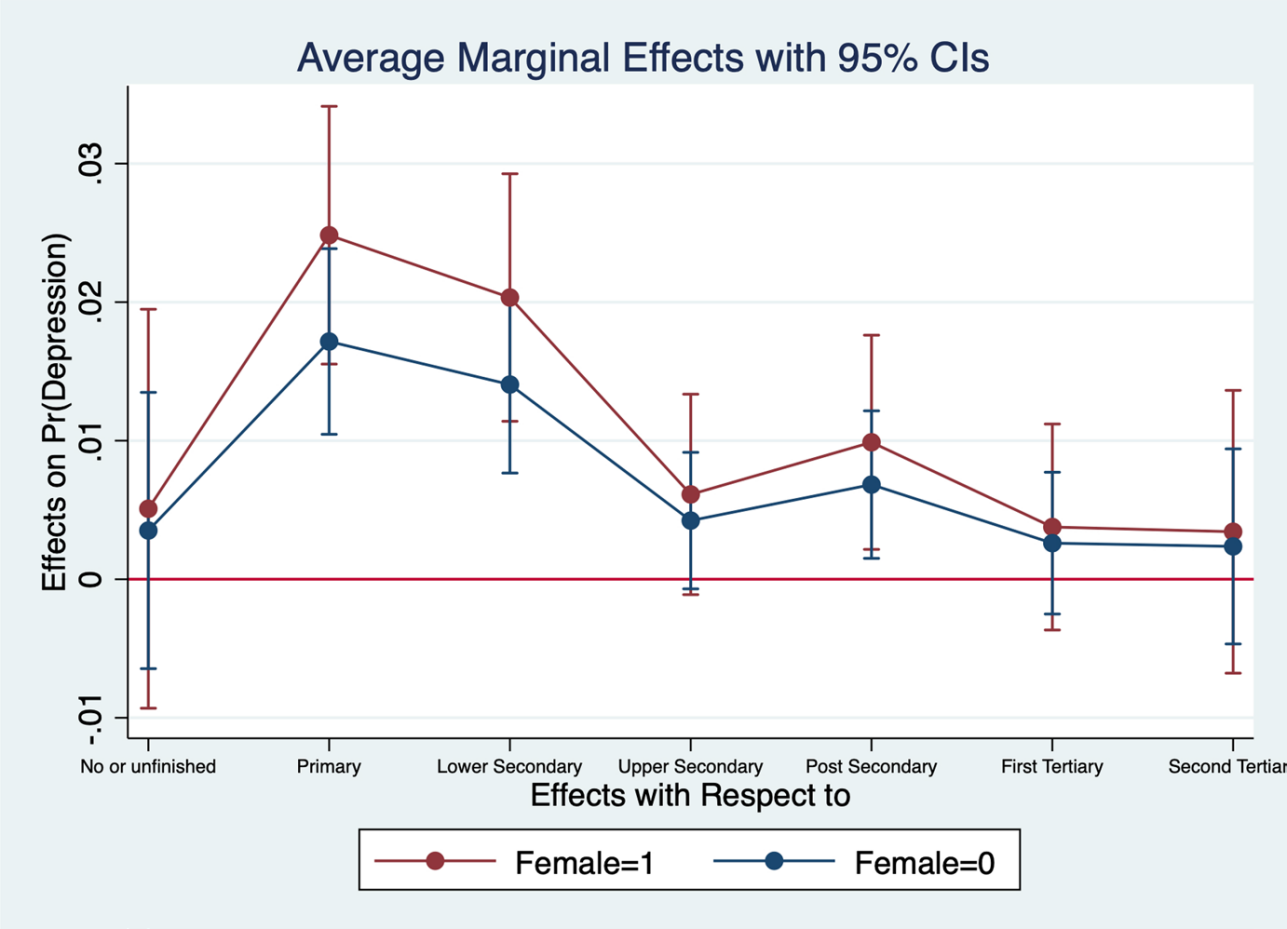
Gendered marginal effects: education classes. Table 3, Column (1) estimate. Average Marginal effect of education status on depression. The Upper Tertiary class of educational status is the omitted benchmark

**Fig. 5:**
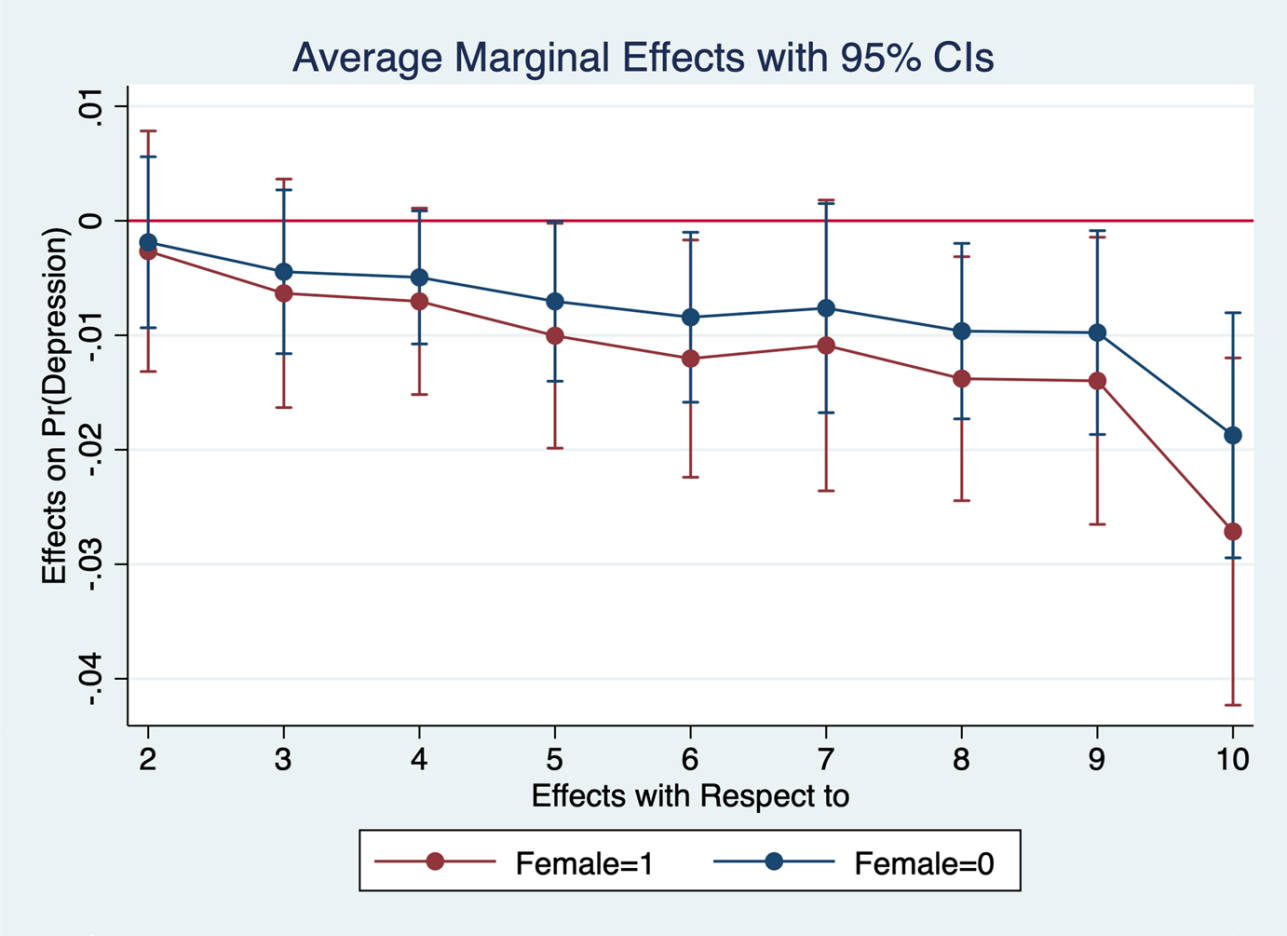
Gendered marginal effects: household income deciles. Table 3, Column (1) estimate. Average Marginal effect of household’s net income on depression. The first (lowest income) class of the household’s net income is the omitted benchmark

**Fig. 6:**
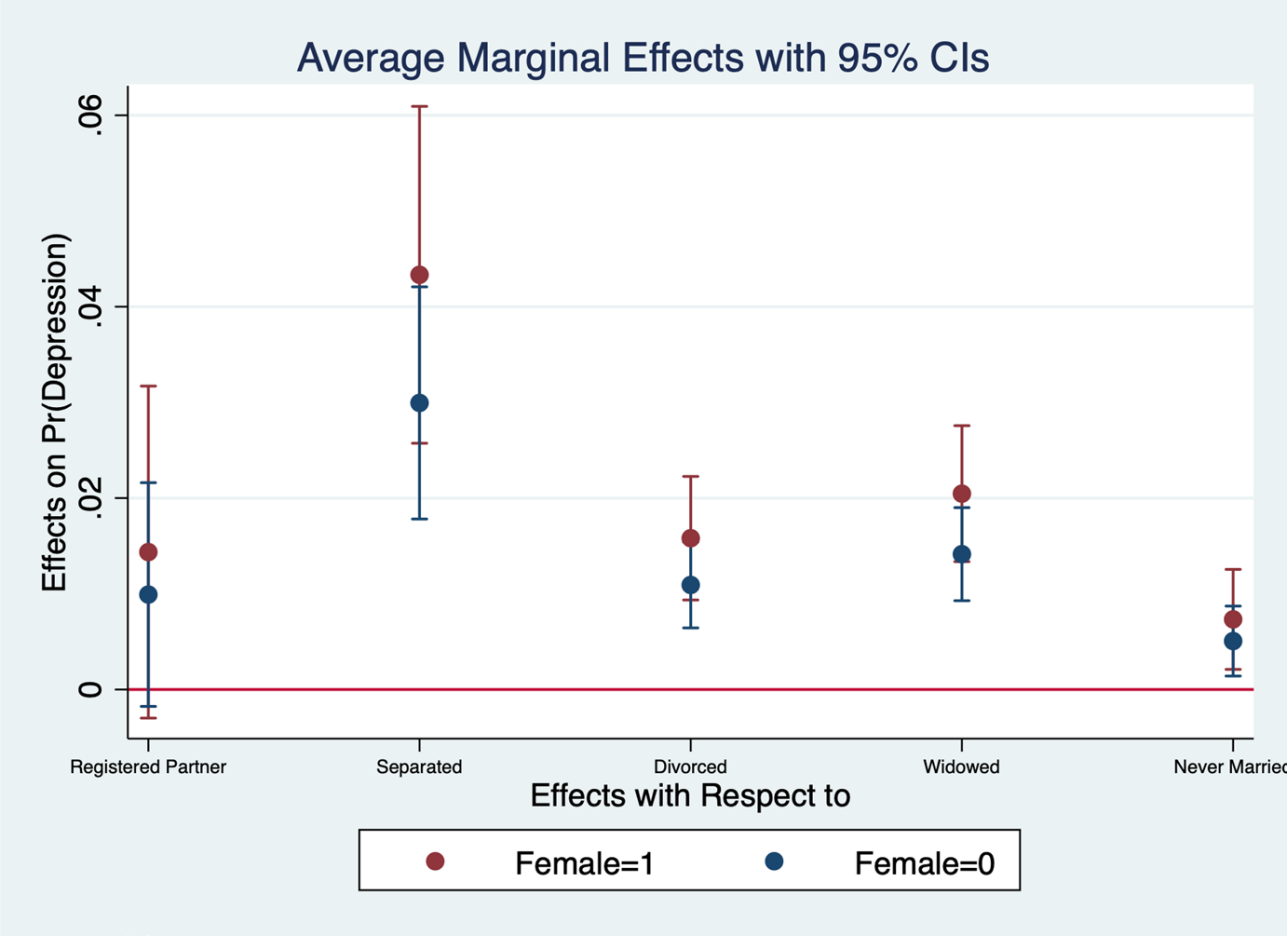
Gendered marginal effects: marital status. Table 3, Column (1) estimate. Average Marginal effect of marital status on depression. The “married” class of marital status is the omitted benchmark

**Fig. 7:**
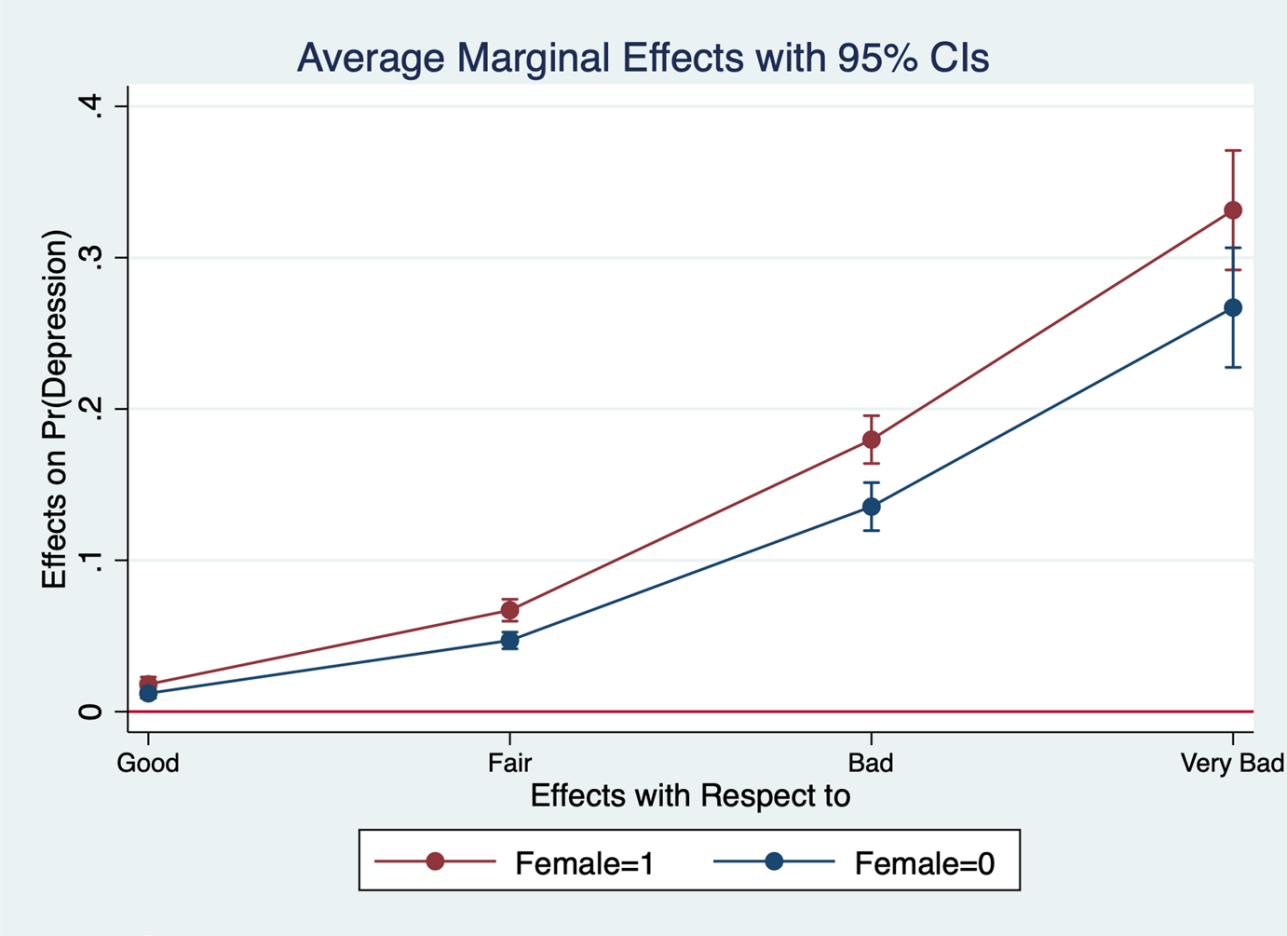
Gendered marginal effects: self-assessed health. Table 3, Column (1) estimate. Average Marginal effect of self health on depression. The “Very Good” class of self.-assessed health is the omitted benchmark

**Fig. 8:**
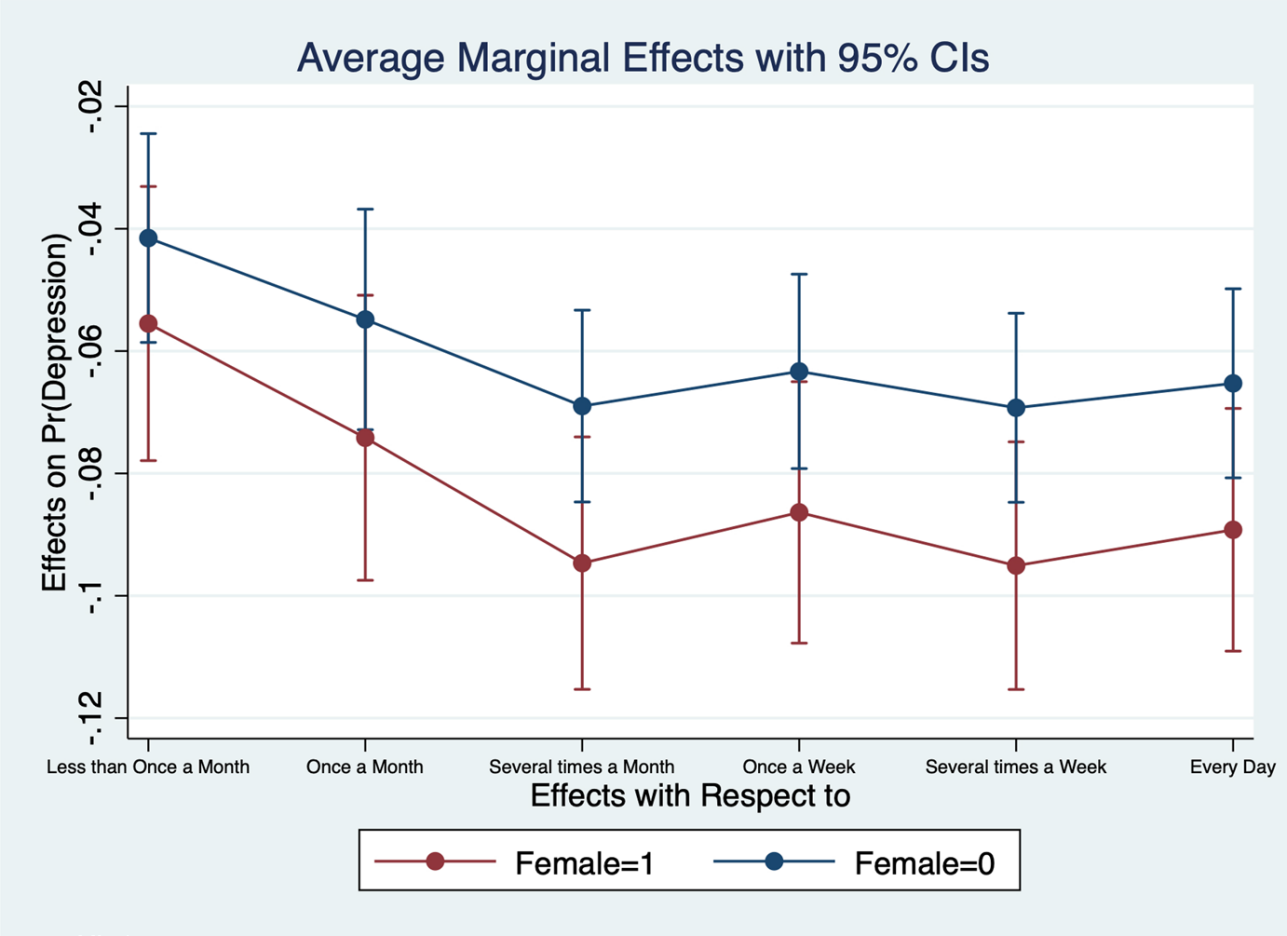
Gendered marginal effects: frequency of social meetings. Table 3, Column (1) estimate. Average Marginal effect of social meeting on depression. The “Never” class of social meeting is the omitted benchmark

**Fig. 9:**
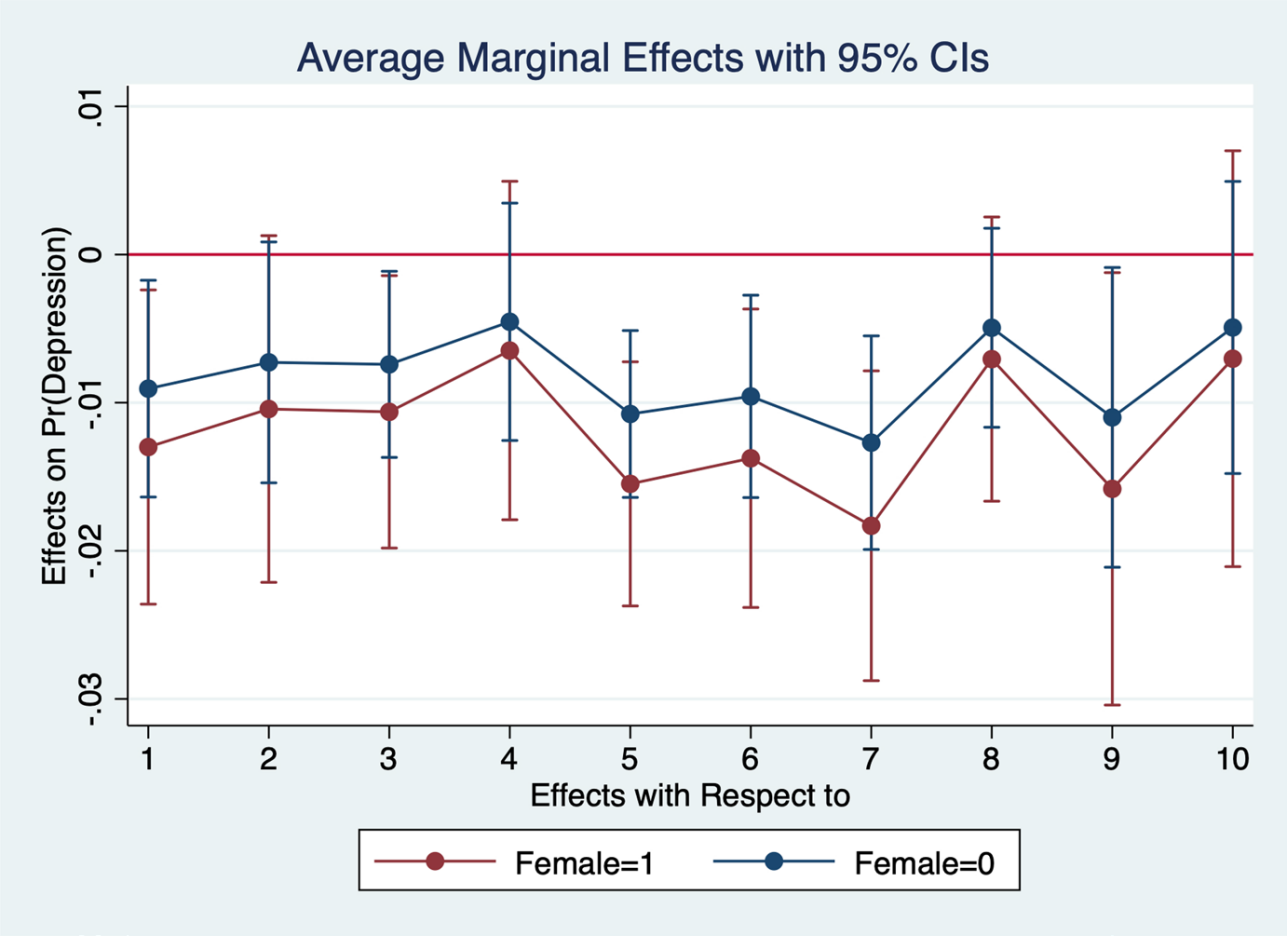
Gendered marginal effects: political orientation. Table 3, Column (1) estimate. Average Marginal effect of Placement on the left right scale on depression. The 0 class (extreme left) is the omitted benchmark

**Fig. 10:**
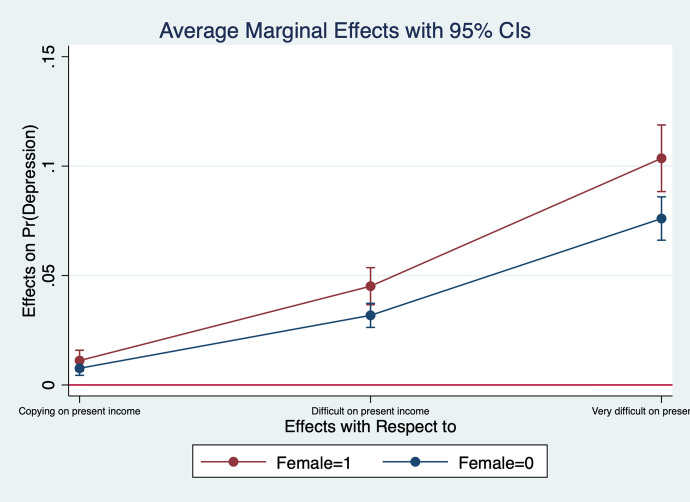
Gendered marginal effects: income satisfaction. Table 3, Column (1) estimate. Average Marginal effect of feelings about income nowadays on depression. The “Living Comfortably on present income” is the omitted benchmark.

Diener et al. (1985) observe that women report higher emotional intensity for the positive/negative events occurring during their lives. In order to test whether the affect intensity rationale accounts for what we observe and comment in section 4 we re-estimate the model by interacting all regressors with gender variables. What we find is that the differences between genders are statistically significant for the variables analysed. In particular, the impact of bad/good news and bad/good states is higher when news and states are interacted with gender dummies. More specifically, states of self-assessed health, age, employment relation, education, social meetings and income have an impact that is stronger in magnitude when interacted with gender dummies (Figs. 2, 3, 4, 5, 6, 7, 8, 9 and 10).

A different way to test the affect intensity rationale is to use the ESS question on whether respondents revert to their previous wellbeing level after a shock. This information measures their resilience.

Resilience is related to one side of affect intensity that is, the dynamics of convergence to the previous wellbeing state in case of negative shocks. Individuals with lower resilience suffer from the negative wellbeing effect of a negative shock for a longer period of time and therefore are more likely to declare a lower level of life satisfaction after a negative shock^2^.

Our estimate with resilience used as the dependent variable shows that the female dummy is negative and significant thereby finding that women are less resilient (Table 9). Among other regressors the effect of income, unemployment and social relationships on resilience is somewhat expected. Monetary resources help to cope with shocks, unemployment has a permanent and negative effect on life satisfaction together with the death of spouse or child (much more than most of the other life shocks where the literature finds hedonic adaptation and therefore resilience, see Clark et al. 2008 and Frijters et al. 2011 among others) and therefore is negatively related with resilience. Social interactions provide consolation and resources to tackle shocks. For these three factors standard (income, employment, relational life) wellbeing policies coincide with what is important also for resilience. The negative effect of being never married on the likelihood of being depressed is apparently puzzling but consistent with the fact that women have more intense relational life and that depression is likely to occur in presence of relational life shocks. Such shocks (ie. health or disability problems of the partner, deterioration of relational quality) are more likely to occur for women with stable relationships. What is less intuitive and interesting to investigate further is the role of age. As is well known a standard result in life satisfaction estimates is that age has a U-shaped effect (Frijters and Beatton 2012). Blanchflower and Oswald (2008) in their survey formulate three interpretations for this quasi-stylized fact: (1) individuals learn to adapt and abandon infeasible aspirations while ageing; (2) cheerful people live longer so that the U-shape is in part explained by a selection effect; (3) the elder compare with more unfortunate outcomes of their peers and revaluate the value of their life. In our paper we find that age helps to revert to the previous happiness level in less time after a negative shock. Interpretation number one (and partially number three) from Blanchflower and Oswald (2008) can help to interpret our findings. The two rationales apply if the developed abilities to adapt better to strengths and weaknesses and to drop unfeasible aspirations also help to be resilient after a shock. This interpretation is supported by very recent evidence from Carstensen et al. (2020) experiment showing that the elders have stronger emotional control than the young and react less adversely to negative situations directing their attention more to emotionally meaningful and positive stimuli. To sum up, our findings on the positive effect of age on resilience and the U-shaped effect of age on life satisfaction are both consistent with the hypothesis that the elders have developed better strategies to cope with emotions and stressing events.

**Table 9:** Resilience and the gender effect

Variables	Resilience
*Female*	−0.122***
	(0.013)
*Age class*	
0–19	−0.026
	(0.032)
20–29	0.018
	(0.022)
30–39	0.001
	(0.013)
50–59	0.028*
	(0.015)
60–69	0.056***
	(0.018)
70–79	0.045*
	(0.026)
80–89	0.090***
	(0.033)
90+	−0.006
	(0.068)
*Employment relation*	
Student	0.041*
	(0.023)
Unemployed, active	−0.124***
	(0.019)
Unemployed, not active	−0.127***
	(0.034)
Retired	−0.032
	(0.020)
*Household’s income*	
2	0.012
	(0.028)
3	0.060***
	(0.022)
4	0.024
	(0.025)
5	0.036
	(0.027)
6	0.087***
	(0.025)
7	0.040
	(0.027)
8	0.094***
	(0.027)
9	0.125***
	(0.029)
10	0.172***
	(0.031)
*Household size*	0.010**
	(0.005)
*Education status*	
No or unfinished	−0.095**
	(0.044)
Primary	−0.181***
	(0.024)
Lower Secondary	−0.159***
	(0.022)
Upper Secondary	−0.063**
	(0.029)
Post-Secondary	−0.057***
	(0.019)
First Level Tertiary	−0.015
	(0.023)
Second Level Tertiary	0.013
	(0.022)
*Marital Status*	
Registered partner	−0.032
	(0.050)
Separated	−0.118
	(0.072)
Divorced	−0.036
	(0.025)
Widowed	−0.065***
	(0.021)
Never Married	−0.065***
	(0.011)
*Self health*	
Good	−0.210***
	(0.019)
Fair	−0.405***
	(0.025)
Bad	−0.578***
	(0.038)
Very Bad	−0.779***
	(0.055)
*Social meeting*	
Less than once a month	0.070
	(0.053)
Once a month	0.128**
	(0.054)
Several times a month	0.200***
	(0.052)
Once a week	0.212***
	(0.053)
Several times a week	0.314***
	(0.053)
Every day	0.364***
	(0.057)
*Placement on left right scale*	
1	0.019
	(0.045)
2	−0.002
	(0.026)
3	−0.001
	(0.031)
4	0.006
	(0.029)
5	0.026
	(0.027)
6	0.004
	(0.030)
7	0.046*
	(0.027)
8	0.064**
	(0.031)
9	0.063
	(0.039)
10	0.059
	(0.039)
*Feeling about Household’s income nowadays*	
Copying on present income	−0.089***
	(0.017)
Difficult on present income	−0.219***
	(0.027)
Very difficult on present income	−0.367***
	(0.028)
*Waves*	
6	0.159***
	(0.026)
*Country dummies*	Yes
*Constant cut1*	−1.105***
	(0.069)
*Constant cut2*	0.013
	(0.063)
*Constant cut3*	0.673***
	(0.071)
*Constant cut4*	2.132***
	(0.092)
*Observations*	63,730
*Pseudo R-Squared*	0.049

Sample survival indicates the marginal effects of the covariates on the survival across waves. Standard errors clustered at country level in parentheses.

*** $$p<0.01$$, ** $$p<0.05$$, * $$p<0.1$$

In order to check whether observable cultural variables can account for the paradox we re-estimate our two base equations for the subsample of Scandinavian countries where the cultural imprinting with the different education of the two sexes may be smaller (Table 6). We find that the paradox persists. More specifically, the gender life satisfaction difference is higher (even though high standard errors in the two Scandinavian/non Scandinavian subsample estimates tell us that the difference is not statistically significant), while the gender depression difference remains the same. This obviously does not imply that the paradox could not be explained by cultural and/or socioeconomic factors but just that the observable cultural and socioeconomic factors accounted for in this sample split do not fully account for it.

## Conclusions

The empirical literature on subjective wellbeing has identified a gender life satisfaction/depression paradox: women are more likely than men to declare the highest level of life satisfaction and, at the same time, more likely to say that they have been depressed in the recent past.

Our empirical analysis aims to shed light on the paradox. First of all, we test the two parts of the paradox jointly and for a large sample of countries in different time periods showing that the paradox is robust.

Second, we find evidence for the affect intensity rationale showing that (positive or negative) events or achievements impact relatively more on life satisfaction of women than men. Third, we more specifically observe that women are less resilient that is, they declare in a significantly higher proportion than men to take more time to absorb negative shocks, even though their lower resilience does not explain all the paradox. Fourth, we wonder whether the gender paradox disappears when we test it in subsamples of women presumably having less traditional training in sex roles (younger, more educated, living in Scandinavian countries) and find that it is not the case.

Our findings suggest that policies to address depression need to take into account these gender differences and have interesting implications on at least two dimensions. First, policymakers are interested in understanding the paradox in order to tackle depression with gender differentiated policies in order to avoid its health costs on the government budget and its productivity costs on the economy. Second, companies are interested to understand the gender differentiated mechanisms to stimulate intrinsic motivations and avoid productivity losses of their workers.

Our findings suggest that a more detailed knowledge of the mechanisms ruling the gender life satisfaction/depression paradox can be of great help in finding the right strategies to pursue gender equality goals (as from goal 5 of the UN Sustainable Development Goals) and to reduce health and productivity costs of depression^3^. If strongest affect intensity and lower resilience are at the root of the gender paradox strategies aimed to increase women resilience to shocks (i.e. stronger support after adversity, trauma, tragedy and threats) can significantly contribute to achieve this goal. A likely major application could be allowing for gender differences in corporate soft skill training courses that are becoming more and more common. Given that resilience is a soft skill and that soft skills contribute significantly to wage skill differentials (Balcar 2014), gender calibrated soft skill training courses can contribute to bridge the gender wage gap contributing to the SDG goal of gender equality.

## Appendix 1

Note that each table also presents the specific country effects (Table 10, 11, 12, 13 and 14).

**Table 10:** Stability of the Gender paradox across Scandinavian Countries

Variables	Scandinavian Countries		Not Scandinavian Countries	
	(1)	(2)	(3)	(4)
	Depression	Life Sat.	Depression	Life Sat.
*Female*	0.168***	0.156***	0.179***	0.109***
	(0.052)	(0.034)	(0.034)	(0.011)
*Age class*				
0–19	0.130	0.002	0.150**	0.164***
	(0.153)	(0.066)	(0.064)	(0.022)
20–29	0.116	0.162***	0.061*	0.127***
	(0.088)	(0.027)	(0.032)	(0.017)
30–39	−0.052	0.098***	0.039	0.074***
	(0.060)	(0.018)	(0.033)	(0.011)
50–59	−0.139***	0.055***	−0.060***	0.025*
	(0.054)	(0.012)	(0.021)	(0.014)
60–69	−0.380***	0.191***	−0.145***	0.105***
	(0.130)	(0.051)	(0.030)	(0.028)
70–79	−0.598***	0.268***	−0.198***	0.179***
	(0.191)	(0.056)	(0.038)	(0.033)
80-89	−0.320**	0.355***	−0.227***	0.227***
	(0.130)	(0.087)	(0.045)	(0.044)
90+	−0.225	0.555***	−0.213**	0.197***
	(0.396)	(0.115)	(0.089)	(0.042)
*Employment relation*				
Student	0.034	0.024	−0.055*	0.045***
	(0.090)	(0.034)	(0.033)	(0.015)
Unemployed, active	−0.018	−0.202***	0.153***	−0.173***
	(0.069)	(0.032)	(0.036)	(0.023)
Unemployed, not active	0.190	−0.223***	0.172***	−0.132***
	(0.116)	(0.051)	(0.052)	(0.024)
Retired	0.066	0.061*	−0.047	0.062***
	(0.106)	(0.037)	(0.031)	(0.013)
*Household’s income*				
2	0.042	−0.015	−0.010	0.022
	(0.043)	(0.036)	(0.042)	(0.015)
3	0.077	0.044**	−0.042	0.025
	(0.070)	(0.020)	(0.040)	(0.019)
4	0.020	0.017	−0.042	0.020
	(0.065)	(0.037)	(0.033)	(0.020)
5	0.006	0.054	−0.060	0.028
	(0.087)	(0.038)	(0.041)	(0.023)
6	0.033	0.064*	−0.080*	0.041*
	(0.106)	(0.033)	(0.042)	(0.021)
7	−0.006	0.104*	−0.063	0.058**
	(0.159)	(0.055)	(0.051)	(0.023)
8	−0.002	0.085*	−0.094**	0.054**
	(0.109)	(0.047)	(0.044)	(0.024)
9	−0.003	0.075	−0.089	0.076***
	(0.104)	(0.049)	(0.055)	(0.028)
10	−0.192	0.096*	−0.191***	0.089***
	(0.177)	(0.050)	(0.070)	(0.027)
*Household size*	−0.029	0.064***	−0.012	0.041***
	(0.025)	(0.006)	(0.010)	(0.005)
*Education status*				
No or unfinished	−0.048	0.207***	0.051	0.053
	(0.037)	(0.050)	(0.065)	(0.057)
Primary	−0.129	0.210***	0.233***	0.055*
	(0.122)	(0.054)	(0.036)	(0.030)
Lower Secondary	0.059	0.204***	0.166***	−0.025
	(0.139)	(0.023)	(0.037)	(0.027)
Upper Secondary	−0.061	0.196***	0.058*	−0.012
	(0.083)	(0.038)	(0.030)	(0.015)
Post−Secondary	−0.095***	0.136***	0.093***	−0.028*
	(0.027)	(0.028)	(0.033)	(0.015)
First Level Tertiary	−0.127	0.131***	0.050*	0.010
	(0.078)	(0.025)	(0.030)	(0.015)
Second Level Tertiary	−0.024	0.031**	0.025	0.007
	(0.131)	(0.013)	(0.042)	(0.014)
*Marital Status*				
Registered partner	−0.033	−0.185***	0.110	−0.163***
	(0.242)	(0.037)	(0.070)	(0.043)
Separated	0.502***	−0.621***	0.281***	−0.433***
	(0.129)	(0.027)	(0.073)	(0.030)
Divorced	−0.012	−0.236***	0.128***	−0.261***
	(0.062)	(0.032)	(0.026)	(0.017)
Widowed	0.116	−0.362***	0.178***	−0.307***
	(0.125)	(0.038)	(0.029)	(0.032)
Never Married	−0.011	−0.322***	0.041**	−0.264***
	(0.067)	(0.011)	(0.021)	(0.021)
*Self health*				
Good	0.212**	−0.330***	0.201***	−0.317***
	(0.092)	(0.034)	(0.033)	(0.020)
Fair	0.700***	−0.679***	0.558***	−0.588***
	(0.033)	(0.064)	(0.038)	(0.028)
Bad	1.160***	−1.031***	1.085***	−0.903***
	(0.093)	(0.099)	(0.049)	(0.038)
Very Bad	1.758***	−1.257***	1.558***	−1.248***
	(0.071)	(0.191)	(0.079)	(0.041)
*Social meeting*				
Less than once a month	−0.229	0.393	−0.295***	0.190***
	(0.537)	(0.258)	(0.055)	(0.040)
Once a month	−0.400	0.531**	−0.418***	0.348***
	(0.513)	(0.257)	(0.062)	(0.041)
Several times a month	−0.621	0.655**	−0.565***	0.443***
	(0.503)	(0.257)	(0.049)	(0.038)
Once a week	−0.590	0.671***	−0.494***	0.479***
	(0.472)	(0.246)	(0.053)	(0.041)
Several times a week	−0.666	0.800***	−0.562***	0.595***
	(0.454)	(0.250)	(0.049)	(0.039)
Every day	−0.612	0.896***	−0.518***	0.735***
	(0.444)	(0.241)	(0.049)	(0.039)
*Placement on left right scale*				
1	−0.060	−0.090*	−0.106***	−0.101***
	(0.220)	(0.054)	(0.037)	(0.022)
2	−0.149	−0.175***	−0.072	−0.149***
	(0.148)	(0.037)	(0.046)	(0.026)
3	−0.128	−0.235***	−0.074**	−0.162***
	(0.132)	(0.037)	(0.034)	(0.029)
4	−0.084	−0.264***	−0.048	−0.175***
	(0.171)	(0.042)	(0.041)	(0.024)
5	−0.167	−0.109**	−0.111***	−0.072**
	(0.106)	(0.047)	(0.030)	(0.029)
6	−0.101	−0.184***	−0.104***	−0.114***
	(0.115)	(0.036)	(0.039)	(0.034)
7	−0.345***	−0.154***	−0.105***	−0.095***
	(0.114)	(0.046)	(0.041)	(0.032)
8	−0.078	−0.033	−0.050	0.028
	(0.120)	(0.028)	(0.037)	(0.037)
9	−0.316	0.059*	−0.094	0.104***
	(0.243)	(0.031)	(0.059)	(0.034)
10	−0.278***	0.226***	−0.032	0.234***
	(0.081)	(0.062)	(0.054)	(0.047)
*Feeling about Household’s income nowadays*				
Copying on present income	0.116*	−0.241***	0.093***	−0.236***
	(0.063)	(0.024)	(0.024)	(0.018)
Difficult on present income	0.321***	−0.512***	0.331***	−0.514***
	(0.060)	(0.043)	(0.035)	(0.035)
Very difficult on present income	0.615***	−0.733***	0.621***	−0.803***
	(0.095)	(0.036)	(0.044)	(0.043)
*Waves*				
4		0.030		0.022
		(0.032)		(0.024)
5		0.035		0.072**
		(0.044)		(0.030)
6	−0.037***	0.157***	−0.092**	0.102***
	(0.011)	(0.054)	(0.042)	(0.033)
7	−0.058	0.084	−0.146***	0.105***
	(0.061)	(0.059)	(0.041)	(0.031)
8		0.148*		0.180***
		(0.076)		(0.032)
*Country dummies*	Yes	Yes	Yes	Yes
*Constant*	−1.420***		−1.335***	
	(0.348)		(0.067)	
*Observations*	19,079	36,614	73,503	161,268
*Pseudo R-Squared*	0.165	0.0597	0.166	0.0756
Country effects across Scandinavian countries				
*Country*				
Austria			−0.325***	0.226***
			(0.034)	(0.035)
Belgium			−0.119***	0.377***
			(0.033)	(0.022)
Bulgaria			−0.298***	−0.391***
			(0.026)	(0.028)
Switzerland			−0.220***	0.514***
			(0.038)	(0.025)
Cyprus			−0.256***	0.328***
			(0.027)	(0.030)
Czech Republic			0.179***	0.010
			(0.029)	(0.021)
Germany			−0.174***	0.369***
			(0.034)	(0.020)
Denmark	−0.122***	0.375***		
	(0.032)	(0.018)		
Estonia			−0.260***	0.220***
			(0.023)	(0.019)
Spain			−0.174***	0.419***
			(0.029)	(0.019)
Finland	−0.456***	0.315***		
	(0.028)	(0.014)		
France			−0.082***	0.093***
			(0.031)	(0.021)
United Kingdom			−0.200***	0.300***
			(0.031)	(0.034)
Greece				−0.231***
				(0.040)
Croatia				−0.076***
				(0.026)
Hungary			0.046*	0.011
			(0.026)	(0.020)
Ireland			−0.317***	0.166***
			(0.028)	(0.030)
Israel			−0.148***	0.285***
			(0.022)	(0.029)
Iceland	0.003	0.213***		
	(0.080)	(0.026)		
Italy			−0.238***	0.112***
			(0.017)	(0.017)
Lithuania			−0.331***	−0.046*
			(0.028)	(0.027)
Latvia			−0.213***	0.014
			(0.051)	(0.035)
Netherlands			−0.349***	0.380***
			(0.035)	(0.022)
Norway	−0.321***	0.178***		
	(0.046)	(0.006)		
Poland			0.013	0.341***
			(0.030)	(0.019)
Portugal			−0.263***	−0.023
			(0.027)	(0.026)
Romania				−0.040
				(0.033)
Russian Federation			−0.124***	−0.001
			(0.036)	(0.024)
Slovenia			−0.457***	0.220***
			(0.028)	(0.020)
Slovakia			−0.170***	0.057***
			(0.033)	(0.020)
Turkey				−0.620***
				(0.060)
Ukraine			−0.100***	−0.117***
			(0.026)	(0.025)
Kosovo			−0.061***	−0.217***
			(0.017)	(0.010)
*Observations*	19,079	36,614	73,503	161,268
*Pseudo R-Squared*	0.165	0.0597	0.166	0.0756

(1) the dependent variable is “Depression”, (2) the dependent variable is “Life Satisfaction” by considering the sample with Scandinavian countries. (3) the dependent variable is “Depression”, (4) the dependent variable is “Life Satisfaction” by considering the sample without Scandinavian countries. Sample survival indicates the marginal effects of the covariates on the survival across waves. Standard errors clustered at country level in parentheses

*** $$p<0.01$$, ** $$p<0.05$$, * $$p<0.1$$.

Country coefficients are those of the corresponding Table 1. Denmark is the omitted benchmark for specifications (1) and (2), while Albania is the omitted benchmark for specifications (3) and (4). Note that: (1) the dependent variable is “Depression”, (2) the dependent variable is “Life Satisfaction” by considering the sample with Scandinavian countries. (3) the dependent variable is “Depression” , (4) the dependent variable is “Life Satisfaction” by considering the sample without Scandinavian countries. Sample survival indicates the marginal effects of the covariates on the survival across waves. Standard errors clustered at country level in parentheses

**Table 11:** Stability of the Gender paradox across Age classes and Educational levels

Variables	Age between 0–59		Age between 60–90+		Low level of Educ		High level of Educ	
	(1)	(2)	(3)	(4)	(5)	(6)	(7)	(8)
	Depression	Life Sat.	Depression	Life Sat.	Depression	Life Sat.	Depression	Life Sat.
*Female*	0.184***	0.129***	0.168***	0.092***	0.204***	0.097***	0.147***	0.132***
	(0.033)	(0.011)	(0.036)	(0.012)	(0.034)	(0.011)	(0.033)	(0.011)
*Age class*								
0-19	0.148**	0.133***			0.078	0.148***	0.169*	0.156***
	(0.065)	(0.026)			(0.091)	(0.030)	(0.095)	(0.038)
20-29	0.077**	0.136***			0.099*	0.104***	0.062*	0.154***
	(0.030)	(0.016)			(0.058)	(0.022)	(0.036)	(0.020)
30-39	0.038	0.080***			0.041	0.049***	0.030	0.096***
	(0.030)	(0.010)			(0.046)	(0.018)	(0.033)	(0.015)
50-59	−0.081***	0.037***	−0.115	−0.037	−0.061*	0.048**	−0.104***	0.015
	(0.020)	(0.013)	(0.087)	(0.045)	(0.032)	(0.022)	(0.023)	(0.013)
60-69	−0.238***	0.077	−0.129	−0.026	−0.194***	0.125***	−0.155***	0.092***
	(0.085)	(0.054)	(0.088)	(0.047)	(0.062)	(0.037)	(0.051)	(0.021)
70-79			−0.179**	0.037	−0.224***	0.200***	−0.278***	0.169***
			(0.085)	(0.044)	(0.068)	(0.044)	(0.088)	(0.022)
80-89			−0.171*	0.084*	−0.283***	0.248***	−0.199*	0.288***
			(0.103)	(0.047)	(0.066)	(0.054)	(0.116)	(0.037)
90+			−0.146	0.073	−0.399**	0.319***	0.086	0.103
			(0.112)	(0.073)	(0.158)	(0.063)	(0.185)	(0.068)
*Employment relation*								
Student	−0.035	0.036***	0.057	0.052	−0.026	−0.012	−0.024	0.040***
	(0.031)	(0.013)	(0.208)	(0.065)	(0.075)	(0.024)	(0.040)	(0.015)
Unemployed, active	0.134***	−0.180***	0.059	−0.172**	0.109***	−0.156***	0.136**	−0.199***
	(0.033)	(0.017)	(0.088)	(0.067)	(0.022)	(0.024)	(0.056)	(0.023)
Unemployed, not active	0.157***	−0.147***	0.180**	−0.172***	0.170**	−0.145***	0.168**	−0.138***
	(0.051)	(0.026)	(0.089)	(0.042)	(0.068)	(0.032)	(0.073)	(0.032)
Retired	−0.024	0.052**	−0.041	0.075***	−0.008	0.057***	−0.023	0.081***
	(0.053)	(0.024)	(0.038)	(0.011)	(0.045)	(0.020)	(0.049)	(0.016)
*Household’s income*								
2	−0.018	0.019	0.004	0.007	−0.019	−0.008	−0.023	0.051***
	(0.037)	(0.019)	(0.058)	(0.018)	(0.044)	(0.016)	(0.042)	(0.020)
3	−0.079*	0.039*	0.027	0.010	−0.058	0.010	−0.031	0.041*
	(0.048)	(0.020)	(0.056)	(0.021)	(0.045)	(0.021)	(0.052)	(0.025)
4	−0.022	0.040*	−0.079*	−0.012	−0.060	0.007	−0.020	0.033
	(0.039)	(0.020)	(0.043)	(0.026)	(0.041)	(0.020)	(0.043)	(0.024)
5	−0.076	0.038*	−0.023	0.027	−0.115***	0.019	−0.015	0.051**
	(0.051)	(0.021)	(0.051)	(0.027)	(0.045)	(0.024)	(0.069)	(0.021)
6	−0.098**	0.060***	−0.037	0.026	−0.138***	0.044*	−0.022	0.051*
	(0.047)	(0.020)	(0.066)	(0.027)	(0.051)	(0.023)	(0.049)	(0.026)
7	−0.117**	0.082***	0.042	0.045	−0.028	0.033	−0.063	0.089***
	(0.056)	(0.022)	(0.073)	(0.029)	(0.051)	(0.025)	(0.066)	(0.025)
8	−0.132***	0.087***	0.020	0.013	−0.044	0.037	−0.062	0.083***
	(0.050)	(0.024)	(0.077)	(0.030)	(0.042)	(0.035)	(0.059)	(0.025)
9	−0.134**	0.099***	0.028	0.050	−0.073	0.037	−0.067	0.110***
	(0.059)	(0.024)	(0.098)	(0.040)	(0.066)	(0.032)	(0.072)	(0.026)
10	−0.224***	0.118***	−0.188	0.028	−0.143*	0.033	−0.205**	0.139***
	(0.076)	(0.025)	(0.120)	(0.033)	(0.080)	(0.037)	(0.088)	(0.025)
*Household size*	−0.012	0.046***	−0.021	0.039***	−0.015	0.053***	−0.008	0.043***
	(0.009)	(0.004)	(0.019)	(0.010)	(0.012)	(0.006)	(0.011)	(0.005)
*Education status*								
No or unfinished	0.044	0.080*	0.016	0.105				
	(0.061)	(0.042)	(0.080)	(0.065)				
Primary	0.249***	0.119**	0.134**	0.066**	0.170***	0.040**		
	(0.047)	(0.046)	(0.068)	(0.031)	(0.049)	(0.019)		
Lower Secondary	0.174***	0.014	0.093	0.009	0.111***	−0.006		
	(0.047)	(0.033)	(0.084)	(0.028)	(0.034)	(0.012)		
Upper Secondary	0.075**	0.012	−0.032	0.029				
	(0.035)	(0.022)	(0.074)	(0.029)				
Post-Secondary	0.106***	−0.006	−0.024	0.009			0.073***	−0.011
	(0.035)	(0.020)	(0.086)	(0.025)			(0.028)	(0.020)
First Level Tertiary	0.036	0.032*	0.003	0.022			0.027	0.030*
	(0.031)	(0.017)	(0.066)	(0.020)			(0.027)	(0.016)
Second Level Tertiary	0.025	0.004	0.043	−0.013			0.030	0.009
	(0.040)	(0.012)	(0.070)	(0.024)			(0.038)	(0.011)
*Marital Status*								
Registered partner	0.100*	−0.165***	0.095	−0.162***	0.079	−0.156***	0.104	−0.115***
	(0.057)	(0.036)	(0.210)	(0.060)	(0.098)	(0.053)	(0.081)	(0.034)
Separated	0.343***	−0.464***	0.224	−0.457***	0.430***	−0.459***	0.071	−0.441***
	(0.079)	(0.039)	(0.161)	(0.056)	(0.091)	(0.072)	(0.166)	(0.041)
Divorced	0.108***	−0.258***	0.122**	−0.247***	0.135***	−0.269***	0.120***	−0.257***
	(0.029)	(0.017)	(0.050)	(0.021)	(0.030)	(0.019)	(0.031)	(0.019)
Widowed	0.241***	−0.380***	0.162***	−0.289***	0.165***	−0.308***	0.182***	−0.323***
	(0.046)	(0.034)	(0.032)	(0.030)	(0.035)	(0.033)	(0.052)	(0.030)
Never Married	0.041	−0.271***	0.012	−0.269***	0.064**	−0.264***	0.037	−0.286***
	(0.025)	(0.019)	(0.042)	(0.025)	(0.027)	(0.024)	(0.028)	(0.019)
*Self health*								
Good	0.198***	−0.336***	0.196***	−0.286***	0.153***	−0.306***	0.252***	−0.329***
	(0.033)	(0.018)	(0.048)	(0.023)	(0.035)	(0.020)	(0.042)	(0.018)
Fair	0.604***	−0.615***	0.480***	−0.550***	0.498***	−0.553***	0.635***	−0.639***
	(0.038)	(0.027)	(0.060)	(0.030)	(0.041)	(0.028)	(0.044)	(0.026)
Bad	1.122***	−0.929***	1.021***	−0.861***	1.038***	−0.878***	1.129***	−0.947***
	(0.053)	(0.042)	(0.054)	(0.036)	(0.047)	(0.038)	(0.064)	(0.032)
Very Bad	1.558***	−1.258***	1.545***	−1.198***	1.642***	−1.255***	1.384***	−1.233***
	(0.087)	(0.058)	(0.073)	(0.051)	(0.081)	(0.044)	(0.095)	(0.062)
*Social meeting*								
Less than once a month	−0.259***	0.190***	−0.327***	0.217***	−0.220***	0.174***	−0.392***	0.278***
	(0.067)	(0.048)	(0.065)	(0.046)	(0.071)	(0.041)	(0.067)	(0.068)
Once a month	−0.444***	0.361***	−0.346***	0.354***	−0.347***	0.315***	−0.511***	0.441***
	(0.072)	(0.054)	(0.079)	(0.045)	(0.082)	(0.035)	(0.068)	(0.077)
Several times a month	−0.584***	0.461***	−0.527***	0.451***	−0.463***	0.413***	−0.701***	0.546***
	(0.067)	(0.050)	(0.062)	(0.045)	(0.066)	(0.037)	(0.061)	(0.072)
Once a week	−0.541***	0.502***	−0.415***	0.462***	−0.399***	0.441***	−0.638***	0.587***
	(0.069)	(0.050)	(0.052)	(0.048)	(0.059)	(0.042)	(0.070)	(0.075)
Several times a week	−0.606***	0.622***	−0.505***	0.579***	−0.455***	0.563***	−0.701***	0.699***
	(0.063)	(0.051)	(0.058)	(0.043)	(0.062)	(0.038)	(0.063)	(0.074)
Every day	−0.564***	0.751***	−0.449***	0.713***	−0.413***	0.689***	−0.664***	0.835***
	(0.061)	(0.053)	(0.060)	(0.040)	(0.064)	(0.038)	(0.068)	(0.072)
*Placement on left right scale*								
1	−0.098*	−0.085***	−0.096*	−0.124***	−0.001	−0.092***	−0.250***	−0.095***
	(0.059)	(0.031)	(0.051)	(0.032)	(0.054)	(0.033)	(0.082)	(0.029)
2	−0.086*	−0.137***	−0.062	−0.181***	−0.118**	−0.167***	−0.071	−0.122***
	(0.048)	(0.031)	(0.071)	(0.038)	(0.059)	(0.036)	(0.059)	(0.028)
3	−0.082**	−0.155***	−0.074	−0.202***	−0.110***	−0.166***	−0.063	−0.161***
	(0.041)	(0.033)	(0.059)	(0.040)	(0.033)	(0.033)	(0.054)	(0.035)
4	−0.083*	−0.173***	0.028	−0.219***	−0.112***	−0.171***	−0.031	−0.175***
	(0.050)	(0.030)	(0.059)	(0.038)	(0.042)	(0.034)	(0.052)	(0.028)
5	−0.118***	−0.064*	−0.113**	−0.108***	−0.146***	−0.067**	−0.117***	−0.069**
	(0.038)	(0.033)	(0.053)	(0.037)	(0.036)	(0.033)	(0.037)	(0.034)
6	−0.101***	−0.119***	−0.089	−0.143***	−0.139***	−0.106***	−0.083**	−0.110***
	(0.036)	(0.034)	(0.074)	(0.041)	(0.052)	(0.038)	(0.034)	(0.035)
7	−0.153***	−0.098***	−0.103*	−0.120***	−0.180***	−0.081**	−0.121***	−0.089**
	(0.052)	(0.036)	(0.058)	(0.045)	(0.052)	(0.035)	(0.041)	(0.039)
8	−0.065	0.034	−0.012	−0.021	−0.117**	0.009	−0.034	0.045
	(0.046)	(0.039)	(0.067)	(0.044)	(0.046)	(0.037)	(0.043)	(0.039)
9	−0.151**	0.114***	−0.050	0.059	−0.127	0.087**	−0.137*	0.112***
	(0.072)	(0.043)	(0.098)	(0.052)	(0.097)	(0.039)	(0.084)	(0.040)
10	−0.093	0.210***	0.001	0.270***	−0.090	0.243***	−0.079	0.229***
	(0.063)	(0.057)	(0.066)	(0.044)	(0.060)	(0.048)	(0.063)	(0.057)
*Feeling about Household’s income nowadays*								
Copying on present income	0.093***	−0.229***	0.105***	−0.244***	0.079*	−0.243***	0.124***	−0.237***
	(0.028)	(0.013)	(0.038)	(0.020)	(0.042)	(0.016)	(0.025)	(0.016)
Difficult on present income	0.321***	−0.503***	0.340***	−0.531***	0.341***	−0.527***	0.327***	−0.514***
	(0.034)	(0.028)	(0.058)	(0.036)	(0.050)	(0.035)	(0.030)	(0.029)
Very difficult on present income	0.621***	−0.802***	0.622***	−0.779***	0.614***	−0.815***	0.638***	−0.793***
	(0.051)	(0.040)	(0.055)	(0.041)	(0.049)	(0.047)	(0.058)	(0.039)
*Waves*								
4		0.031		−0.003		0.015		0.023
		(0.021)		(0.029)		(0.034)		(0.022)
5		0.072***		0.054*		0.073*		0.063***
		(0.027)		(0.031)		(0.042)		(0.021)
6	−0.099**	0.111***	−0.067	0.110***	−0.083	0.123**	−0.103***	0.105***
	(0.044)	(0.031)	(0.044)	(0.031)	(0.055)	(0.050)	(0.034)	(0.022)
7	−0.144***	0.116***	−0.137***	0.066**	−0.158***	0.089**	−0.122***	0.104***
	(0.043)	(0.030)	(0.036)	(0.033)	(0.061)	(0.042)	(0.035)	(0.020)
8		0.182***		0.158***		0.170***		0.173***
		(0.033)		(0.031)		(0.045)		(0.027)
*Constant*	−1.271***		−1.342***		−1.371***		−1.131***	
	(0.074)		(0.174)		(0.109)		(0.083)	
*Observations*	63,420	135,803	29,597	62,515	36,479	75,448	46,706	99,401
*Pseudo R-Squared*	0.167	0.0777	0.203	0.0903	0.181	0.0806	0.157	0.0843
Country effects across Age classes and Educational levels								
*Country*								
Austria	−0.339***	0.266***	−0.296***	0.139***	−0.209***	0.183***	−0.433***	0.482***
	(0.044)	(0.028)	(0.043)	(0.044)	(0.042)	(0.032)	(0.045)	(0.024)
Belgium	−0.112***	0.385***	−0.146***	0.384***	−0.050	0.359***	−0.224***	0.466***
	(0.037)	(0.018)	(0.045)	(0.036)	(0.035)	(0.026)	(0.039)	(0.020)
Bulgaria	−0.244***	−0.292***	−0.325***	−0.577***	−0.295***	−0.624***	−0.401***	−0.257***
	(0.029)	(0.022)	(0.025)	(0.033)	(0.031)	(0.033)	(0.035)	(0.020)
Switzerland	−0.204***	0.510***	−0.298***	0.563***	−0.127***	0.435***	−0.370***	0.657***
	(0.040)	(0.020)	(0.054)	(0.040)	(0.041)	(0.030)	(0.045)	(0.024)
Cyprus	−0.204***	0.306***	−0.345***	0.348***	−0.239***	0.333***	−0.179***	0.296***
	(0.031)	(0.024)	(0.031)	(0.032)	(0.023)	(0.028)	(0.019)	(0.016)
Czech Republic	0.164***	−0.014	0.225***	0.027	0.280***	−0.048*	0.049	0.106***
	(0.036)	(0.019)	(0.034)	(0.032)	(0.031)	(0.029)	(0.036)	(0.018)
Germany	−0.164***	0.387***	−0.244***	0.342***	−0.110***	0.270***	−0.270***	0.526***
	(0.039)	(0.018)	(0.045)	(0.035)	(0.038)	(0.026)	(0.042)	(0.020)
Denmark	−0.264***	0.646***	−0.500***	0.716***	−0.232***	0.615***	−0.432***	0.751***
	(0.038)	(0.024)	(0.056)	(0.042)	(0.042)	(0.031)	(0.042)	(0.029)
Estonia	−0.293***	0.289***	−0.223***	0.079**	−0.111***	0.018	−0.411***	0.381***
	(0.029)	(0.015)	(0.034)	(0.036)	(0.025)	(0.024)	(0.037)	(0.016)
Spain	−0.224***	0.472***	−0.062	0.317***	−0.061*	0.401***	−0.341***	0.449***
	(0.033)	(0.017)	(0.045)	(0.028)	(0.033)	(0.026)	(0.037)	(0.020)
Finland	−0.600***	0.672***	−0.729***	0.527***	−0.650***	0.478***	−0.712***	0.777***
	(0.037)	(0.026)	(0.038)	(0.046)	(0.033)	(0.032)	(0.034)	(0.022)
France	−0.094**	0.158***	−0.071*	−0.047	0.029	0.014	−0.225***	0.211***
	(0.037)	(0.019)	(0.041)	(0.034)	(0.034)	(0.028)	(0.038)	(0.020)
United Kingdom	−0.176***	0.256***	−0.263***	0.377***	−0.174***	0.262***	−0.275***	0.410***
	(0.039)	(0.024)	(0.045)	(0.045)	(0.034)	(0.030)	(0.035)	(0.020)
Greece		−0.228***		−0.304***		−0.383***		−0.208***
		(0.032)		(0.045)		(0.042)		(0.030)
Croatia		−0.028		−0.190***		−0.198***		0.069***
		(0.023)		(0.034)		(0.034)		(0.024)
Hungary	−0.062**	0.048***	0.204***	−0.091***	0.194***	−0.102***	−0.160***	0.154***
	(0.031)	(0.016)	(0.040)	(0.035)	(0.029)	(0.025)	(0.038)	(0.018)
Ireland	−0.354***	0.125***	−0.263***	0.250***	−0.111***	−0.003	−0.505***	0.263***
	(0.031)	(0.022)	(0.049)	(0.042)	(0.032)	(0.029)	(0.027)	(0.017)
Israel	−0.143***	0.303***	−0.160***	0.265***	−0.029	0.264***	−0.278***	0.438***
	(0.030)	(0.023)	(0.043)	(0.036)	(0.027)	(0.025)	(0.030)	(0.018)
Iceland	−0.209***	0.528***	−0.170***	0.530***	−0.165***	0.461***	−0.235***	0.630***
	(0.023)	(0.020)	(0.040)	(0.038)	(0.029)	(0.029)	(0.029)	(0.023)
Italy	−0.241***	0.156***	−0.237***	0.010	−0.106***	0.039*	−0.402***	0.225***
	(0.019)	(0.016)	(0.028)	(0.030)	(0.017)	(0.020)	(0.025)	(0.018)
Lithuania	−0.304***	0.014	−0.341***	−0.188***	−0.195***	−0.251***	−0.480***	0.069***
	(0.036)	(0.021)	(0.035)	(0.039)	(0.030)	(0.027)	(0.040)	(0.016)
Latvia	−0.262***	0.050	−0.081*	−0.095**	−0.050	−0.074*	−0.440***	0.139***
	(0.054)	(0.034)	(0.048)	(0.038)	(0.062)	(0.042)	(0.046)	(0.030)
Netherlands	−0.369***	0.420***	−0.335***	0.315***	−0.251***	0.319***	−0.485***	0.484***
	(0.037)	(0.017)	(0.050)	(0.038)	(0.038)	(0.028)	(0.043)	(0.023)
Norway	−0.452***	0.482***	−0.738***	0.479***	−0.361***	0.463***	−0.673***	0.549***
	(0.036)	(0.022)	(0.050)	(0.039)	(0.038)	(0.028)	(0.041)	(0.024)
Poland	−0.115***	0.381***	0.240***	0.245***	0.115***	0.231***	−0.124***	0.485***
	(0.034)	(0.016)	(0.034)	(0.030)	(0.038)	(0.022)	(0.033)	(0.019)
Portugal	−0.321***	0.035	−0.188***	−0.133***	−0.167***	−0.016	−0.596***	0.099***
	(0.040)	(0.022)	(0.031)	(0.030)	(0.027)	(0.029)	(0.037)	(0.019)
Romania		−0.040		−0.080**		−0.073**		0.004
		(0.031)		(0.035)		(0.036)		(0.032)
Russian Federatio	−0.159***	0.019	−0.083**	−0.050	−0.136***	−0.028	−0.220***	0.089***
	(0.043)	(0.019)	(0.037)	(0.036)	(0.040)	(0.034)	(0.038)	(0.020)
Sweden	−0.186***	0.324***	−0.291***	0.340***	−0.298***	0.303***	−0.265***	0.396***
	(0.040)	(0.027)	(0.046)	(0.046)	(0.037)	(0.033)	(0.036)	(0.021)
Slovenia	−0.500***	0.317***	−0.401***	0.024	−0.385***	0.063***	−0.564***	0.391***
	(0.032)	(0.017)	(0.039)	(0.030)	(0.037)	(0.023)	(0.034)	(0.019)
Slovakia	−0.117***	0.053***	−0.308***	0.025	−0.158***	−0.016	−0.215***	0.142***
	(0.037)	(0.018)	(0.034)	(0.029)	(0.042)	(0.025)	(0.035)	(0.017)
Turkey		−0.643***		−0.462***				
		(0.039)		(0.069)				
Ukraine	−0.114***	−0.040*	−0.077**	−0.289***	0.033	−0.275***	−0.218***	−0.012
	(0.034)	(0.021)	(0.037)	(0.034)	(0.021)	(0.035)	(0.038)	(0.024)
Kosovo	−0.115***	−0.166***	0.166***	−0.454***	0.028	−0.237***	−0.146***	−0.199***
	(0.015)	(0.009)	(0.044)	(0.023)	(0.025)	(0.016)	(0.018)	(0.009)
*Observations*	63,420	135,803	29,597	62,515	36,479	75,448	46,706	99,401
*Pseudo R-Squared*	0.167	0.0777	0.203	0.0903	0.181	0.0806	0.157	0.0843

(1) the dependent variable is “Depression”, (2) the dependent variable is “Life Satisfaction” by considering the sample with persons aged between 0-59. (3) the dependent variable is “Depression”, (4) the dependent variable is “Life Satisfaction” by considering the sample with persons aged between 60-90+. (5) the dependent variable is “Depression”, (6) the dependent variable is “Life Satisfaction” by considering the sample with persons with a low level of education. (7) the dependent variable is “Depression”, (8) the dependent variable is “Life Satisfaction” by considering the sample with persons with a highlevel of education. Sample survival indicates the marginal effects of the covariates on the survival across waves. Standard errors clustered at country level in parentheses.

*** $$p<0.01$$, ** $$p<0.05$$, * $$p<0.1$$

Country coefficients are those of the corresponding table 3. Albania is the omitted benchmark. Note that: (1) the dependent variable is “Depression”, (2) the dependent variable is “Life Satisfaction” by considering the sample with persons aged between 0-59. (3) the dependent variable is “Depression”, (4) the dependent variable is “Life Satisfaction” by considering the sample with persons aged between 60-90+. (5) the dependent variable is “Depression”, (6) the dependent variable is “Life Satisfaction” by considering the sample with persons with a low level of education. (7) the dependent variable is “Depression”, (8) the dependent variable is “Life Satisfaction” by considering the sample with persons with a high level of education. Sample survival indicates the marginal effects of the covariates on the survival across waves. Standard errors clustered at country level in parentheses

**Table 12:** Stability of the Gender paradox across Self-assessed Health and Geographical Areas

Variables	Not Good Self-assessed Health		Good Self-assessed Health		With Eastern		Without Eastern	
	(1)	(2)	(3)	(4)	(5)	(6)	(7)	(8)
	Depression	Life Sat.	Depression	Life Sat.	Depression	Life Sat.	Depression	Life Sat.
*Female*	0.175***	0.121***	0.179***	0.109***	0.141***	0.122***	0.195***	0.114***
	(0.038)	(0.010)	(0.033)	(0.015)	(0.029)	(0.012)	(0.042)	(0.014)
*Age class*								
0-19	0.074	0.140***	0.285***	0.124***	0.128	0.177***	0.152**	0.116***
	(0.074)	(0.026)	(0.089)	(0.041)	(0.081)	(0.021)	(0.076)	(0.032)
20-29	0.031	0.140***	0.163***	0.094***	0.161***	0.167***	0.031	0.119***
	(0.037)	(0.018)	(0.044)	(0.021)	(0.048)	(0.022)	(0.037)	(0.018)
30-39	−0.013	0.084***	0.106**	0.065***	0.032	0.096***	0.028	0.070***
	(0.034)	(0.011)	(0.046)	(0.019)	(0.052)	(0.010)	(0.038)	(0.013)
50-59	−0.065*	0.022	−0.081***	0.045***	−0.061*	0.027	−0.076***	0.034***
	(0.033)	(0.013)	(0.027)	(0.015)	(0.035)	(0.024)	(0.023)	(0.013)
60-69	−0.129***	0.124***	−0.209***	0.114***	−0.148**	0.107**	−0.192***	0.128***
	(0.038)	(0.026)	(0.045)	(0.026)	(0.062)	(0.044)	(0.037)	(0.029)
70-79	−0.183***	0.224***	−0.268***	0.170***	−0.183**	0.177***	−0.270***	0.204***
	(0.067)	(0.031)	(0.049)	(0.029)	(0.072)	(0.039)	(0.051)	(0.035)
80-89	−0.131*	0.319***	−0.282***	0.205***	−0.240***	0.307***	−0.254***	0.240***
	(0.077)	(0.044)	(0.047)	(0.038)	(0.050)	(0.050)	(0.051)	(0.046)
90+	−0.082	0.295***	−0.289***	0.202***	−0.003	0.136	−0.319***	0.301***
	(0.137)	(0.055)	(0.109)	(0.049)	(0.159)	(0.106)	(0.112)	(0.048)
*Employment relation*								
Student	0.019	0.043***	−0.124*	−0.002	−0.026	0.055***	−0.030	0.033**
	(0.040)	(0.014)	(0.066)	(0.023)	(0.064)	(0.021)	(0.037)	(0.016)
Unemployed, active	0.174***	−0.187***	0.091***	−0.171***	0.109	−0.225***	0.146***	−0.158***
	(0.044)	(0.024)	(0.035)	(0.028)	(0.076)	(0.033)	(0.036)	(0.025)
Unemployed, not active	0.204***	−0.120***	0.134**	−0.175***	0.195***	−0.170***	0.162***	−0.131***
	(0.064)	(0.028)	(0.059)	(0.030)	(0.065)	(0.048)	(0.063)	(0.025)
Retired	−0.052	0.085***	−0.027	0.052***	−0.043	0.083***	−0.028	0.055***
	(0.046)	(0.015)	(0.029)	(0.015)	(0.066)	(0.020)	(0.036)	(0.014)
*Household’s income*								
2	0.010	0.006	−0.019	0.017	−0.066	0.005	0.020	0.018
	(0.056)	(0.020)	(0.044)	(0.018)	(0.060)	(0.028)	(0.046)	(0.015)
3	−0.040	0.023	−0.035	0.019	−0.015	0.019	−0.043	0.030
	(0.065)	(0.023)	(0.036)	(0.024)	(0.045)	(0.028)	(0.049)	(0.021)
4	0.012	0.009	−0.079**	0.016	−0.012	0.006	−0.050	0.026
	(0.055)	(0.025)	(0.032)	(0.020)	(0.044)	(0.025)	(0.035)	(0.022)
5	−0.047	0.014	−0.064	0.039	−0.048	0.053	−0.056	0.027
	(0.060)	(0.023)	(0.041)	(0.028)	(0.070)	(0.035)	(0.044)	(0.023)
6	0.011	0.015	−0.155***	0.074***	−0.043	0.074**	−0.076	0.034*
	(0.061)	(0.022)	(0.048)	(0.026)	(0.048)	(0.037)	(0.049)	(0.020)
7	−0.049	0.040	−0.063	0.095***	−0.049	0.083**	−0.060	0.060**
	(0.071)	(0.025)	(0.064)	(0.028)	(0.062)	(0.040)	(0.060)	(0.025)
8	−0.083	0.039	−0.065	0.084***	0.014	0.081	−0.117**	0.055**
	(0.069)	(0.025)	(0.048)	(0.029)	(0.044)	(0.050)	(0.051)	(0.023)
9	−0.039	0.059**	−0.125**	0.089***	0.008	0.082*	−0.115**	0.074***
	(0.079)	(0.026)	(0.058)	(0.032)	(0.090)	(0.048)	(0.052)	(0.027)
10	−0.173**	0.080***	−0.206**	0.073**	−0.179***	0.098**	−0.202**	0.083***
	(0.078)	(0.027)	(0.088)	(0.035)	(0.067)	(0.044)	(0.081)	(0.026)
*Household size*	−0.013	0.044***	−0.016*	0.051***	−0.008	0.034***	−0.018*	0.049***
	(0.011)	(0.004)	(0.009)	(0.007)	(0.017)	(0.010)	(0.010)	(0.004)
*Education status*								
No or unfinished	−0.009	0.094**	0.082	0.044	−0.019	0.037	0.061	0.098*
	(0.070)	(0.048)	(0.073)	(0.048)	(0.064)	(0.029)	(0.071)	(0.055)
Primary	0.248***	0.086**	0.175***	0.032	0.222**	−0.021	0.195***	0.104***
	(0.054)	(0.034)	(0.054)	(0.026)	(0.113)	(0.070)	(0.039)	(0.030)
Lower Secondary	0.170***	0.016	0.136**	−0.015	0.143*	−0.042	0.162***	0.029
	(0.046)	(0.029)	(0.056)	(0.029)	(0.084)	(0.044)	(0.036)	(0.033)
Upper Secondary	0.096**	0.026	−0.002	−0.006	0.044	−0.043	0.046	0.044*
	(0.044)	(0.021)	(0.045)	(0.022)	(0.046)	(0.030)	(0.032)	(0.024)
Post-Secondary	0.071	0.009	0.073	−0.033*	0.089*	−0.057*	0.066*	0.019
	(0.045)	(0.020)	(0.052)	(0.019)	(0.050)	(0.033)	(0.039)	(0.019)
First Level Tertiary	0.058	0.034**	−0.003	0.011	0.082*	−0.033**	0.010	0.052***
	(0.058)	(0.015)	(0.049)	(0.019)	(0.047)	(0.016)	(0.033)	(0.019)
Second Level Tertiary	0.063	0.011	−0.038	−0.015	−0.009	0.009	0.036	0.006
	(0.054)	(0.010)	(0.056)	(0.023)	(0.070)	(0.025)	(0.047)	(0.012)
*Marital Status*								
Registered partner	0.063	−0.157***	0.108	−0.194***	−0.079	−0.141	0.151*	−0.170***
	(0.090)	(0.032)	(0.093)	(0.058)	(0.100)	(0.092)	(0.078)	(0.034)
Separated	0.296**	−0.489***	0.333**	−0.440***	0.138	−0.390***	0.341***	−0.474***
	(0.131)	(0.051)	(0.129)	(0.057)	(0.158)	(0.139)	(0.070)	(0.032)
Divorced	0.073*	−0.251***	0.130***	−0.255***	0.094*	−0.266***	0.117***	−0.249***
	(0.039)	(0.017)	(0.035)	(0.018)	(0.050)	(0.037)	(0.030)	(0.016)
Widowed	0.278***	−0.347***	0.139***	−0.271***	0.116**	−0.253***	0.205***	−0.342***
	(0.048)	(0.034)	(0.028)	(0.028)	(0.048)	(0.041)	(0.030)	(0.035)
Never Married	0.038	−0.276***	0.030	−0.269***	0.015	−0.298***	0.045***	−0.263***
	(0.029)	(0.018)	(0.027)	(0.024)	(0.053)	(0.039)	(0.017)	(0.018)
*Self health*								
Good	0.191***	−0.345***			0.188***	−0.331***	0.214***	−0.315***
	(0.031)	(0.015)			(0.068)	(0.023)	(0.033)	(0.021)
Fair					0.629***	−0.665***	0.560***	−0.579***
					(0.062)	(0.040)	(0.038)	(0.031)
Bad			0.539***	−0.304***	1.218***	−1.022***	1.060***	−0.889***
			(0.030)	(0.014)	(0.099)	(0.055)	(0.041)	(0.041)
Very Bad			1.022***	−0.636***	1.717***	−1.326***	1.540***	−1.234***
			(0.058)	(0.026)	(0.089)	(0.064)	(0.087)	(0.049)
*Social meeting*								
Less than once a month	−0.289***	0.151**	−0.294***	0.233***	−0.257*	0.224***	−0.303***	0.192***
	(0.084)	(0.070)	(0.063)	(0.040)	(0.138)	(0.066)	(0.057)	(0.048)
Once a month	−0.364***	0.329***	−0.450***	0.365***	−0.367**	0.396***	−0.430***	0.344***
	(0.091)	(0.071)	(0.074)	(0.035)	(0.149)	(0.078)	(0.062)	(0.047)
Several times a month	−0.540***	0.417***	−0.588***	0.480***	−0.485***	0.491***	−0.603***	0.447***
	(0.072)	(0.066)	(0.059)	(0.035)	(0.115)	(0.064)	(0.053)	(0.046)
Once a week	−0.497***	0.457***	−0.498***	0.497***	−0.457***	0.548***	−0.516***	0.470***
	(0.066)	(0.066)	(0.059)	(0.040)	(0.113)	(0.069)	(0.059)	(0.048)
Several times a week	−0.568***	0.576***	−0.567***	0.616***	−0.511***	0.635***	−0.593***	0.601***
	(0.065)	(0.066)	(0.057)	(0.035)	(0.107)	(0.058)	(0.054)	(0.049)
Every day	−0.501***	0.717***	−0.549***	0.721***	−0.558***	0.736***	−0.515***	0.738***
	(0.070)	(0.066)	(0.061)	(0.036)	(0.091)	(0.056)	(0.056)	(0.047)
*Placement on left right scale*								
1	−0.122	−0.110***	−0.071	−0.084***	−0.090	−0.048	−0.095**	−0.126***
	(0.093)	(0.031)	(0.044)	(0.032)	(0.082)	(0.040)	(0.045)	(0.023)
2	−0.012	−0.162***	−0.128*	−0.145***	−0.016	−0.091**	−0.097*	−0.180***
	(0.076)	(0.032)	(0.068)	(0.034)	(0.074)	(0.039)	(0.052)	(0.028)
3	−0.042	−0.192***	−0.106**	−0.146***	−0.048	−0.119**	−0.086**	−0.196***
	(0.064)	(0.037)	(0.044)	(0.026)	(0.062)	(0.054)	(0.038)	(0.030)
4	−0.064	−0.207***	−0.026	−0.163***	−0.060	−0.121**	−0.039	−0.217***
	(0.078)	(0.029)	(0.043)	(0.028)	(0.063)	(0.057)	(0.051)	(0.023)
5	−0.104	−0.097***	−0.122***	−0.052*	−0.074	−0.027	−0.128***	−0.101***
	(0.065)	(0.034)	(0.038)	(0.030)	(0.052)	(0.051)	(0.034)	(0.031)
6	−0.062	−0.161***	−0.136***	−0.065*	−0.036	−0.049	−0.122***	−0.160***
	(0.067)	(0.036)	(0.048)	(0.035)	(0.044)	(0.066)	(0.046)	(0.033)
7	−0.165**	−0.140***	−0.105**	−0.046	−0.073	−0.050	−0.158***	−0.132***
	(0.080)	(0.033)	(0.047)	(0.034)	(0.074)	(0.056)	(0.045)	(0.033)
8	−0.097	0.004	0.002	0.028	−0.006	0.084	−0.064	−0.016
	(0.071)	(0.036)	(0.047)	(0.039)	(0.060)	(0.056)	(0.040)	(0.038)
9	−0.065	0.086**	−0.159**	0.101***	−0.165**	0.159**	−0.078	0.064**
	(0.076)	(0.037)	(0.075)	(0.038)	(0.071)	(0.063)	(0.073)	(0.032)
10	−0.103	0.249***	−0.016	0.211***	−0.091	0.224***	−0.015	0.247***
	(0.069)	(0.052)	(0.061)	(0.046)	(0.084)	(0.058)	(0.069)	(0.055)
*Feeling about Household’s income nowadays*								
Copying on present income	0.066**	−0.249***	0.149***	−0.226***	0.043	−0.226***	0.116***	−0.243***
	(0.033)	(0.014)	(0.034)	(0.019)	(0.044)	(0.022)	(0.024)	(0.017)
Difficult on present income	0.302***	−0.523***	0.379***	−0.510***	0.322***	−0.472***	0.336***	−0.539***
	(0.044)	(0.029)	(0.033)	(0.033)	(0.060)	(0.034)	(0.035)	(0.037)
Very difficult on present income	0.626***	−0.779***	0.660***	−0.814***	0.562***	−0.735***	0.656***	−0.834***
	(0.070)	(0.042)	(0.042)	(0.038)	(0.056)	(0.033)	(0.048)	(0.048)
*Waves*								
4		0.033*		0.004		0.040		0.013
		(0.019)		(0.028)		(0.035)		(0.023)
5		0.061**		0.072**		0.075***		0.058*
		(0.027)		(0.034)		(0.022)		(0.034)
6	−0.085*	0.100***	−0.093**	0.125***	−0.172***	0.059	−0.060	0.126***
	(0.048)	(0.029)	(0.037)	(0.038)	(0.028)	(0.036)	(0.049)	(0.037)
7	−0.175***	0.105***	−0.107**	0.086**	−0.197***	0.065**	−0.115**	0.106***
	(0.036)	(0.025)	(0.043)	(0.041)	(0.046)	(0.027)	(0.048)	(0.038)
8		0.173***		0.172***		0.089***		0.197***
		(0.028)		(0.039)		(0.033)		(0.037)
*Constant*	−1.327***		−0.726***		−1.280***		−1.634***	
	(0.111)		(0.093)		(0.141)		(0.065)	
*Observations*	60,952	128,640	31,630	69,242	24,739	53,579	67,843	144,303
*Pseudo R-Squared*	0.0874	0.0624	0.144	0.0654	0.174	0.0843	0.180	0.0799
Country effects across Self Health and Geographical Area								
*Country*								
Austria	−0.416***	0.263***	−0.234***	0.141***				
	(0.050)	(0.028)	(0.038)	(0.033)				
Belgium	−0.136***	0.345***	−0.122***	0.514***			0.218***	0.153***
	(0.041)	(0.019)	(0.036)	(0.022)			(0.024)	(0.019)
Bulgaria	−0.213***	−0.392***	−0.349***	−0.386***			0.013	−0.619***
	(0.040)	(0.024)	(0.022)	(0.029)			(0.030)	(0.024)
Switzerland	−0.339***	0.536***	−0.054	0.508***	−0.317***	0.454***		
	(0.048)	(0.021)	(0.040)	(0.024)	(0.031)	(0.030)		
Cyprus	−0.322***	0.258***	−0.153***	0.524***			0.068***	0.094***
	(0.035)	(0.025)	(0.024)	(0.026)			(0.023)	(0.012)
Czech Republic	0.135***	−0.048**	0.236***	0.118***	0.121***	−0.047		
	(0.041)	(0.020)	(0.030)	(0.023)	(0.028)	(0.034)		
Germany	−0.168***	0.384***	−0.199***	0.390***			0.160***	0.137***
	(0.044)	(0.018)	(0.037)	(0.022)			(0.025)	(0.024)
Denmark	−0.347***	0.642***	−0.313***	0.766***			0.021	0.443***
	(0.044)	(0.026)	(0.037)	(0.028)			(0.022)	(0.025)
Estonia	−0.284***	0.282***	−0.254***	0.217***			0.063**	−0.012
	(0.036)	(0.018)	(0.026)	(0.022)			(0.025)	(0.022)
Spain	−0.141***	0.392***	−0.214***	0.508***			0.159***	0.187***
	(0.037)	(0.019)	(0.031)	(0.021)			(0.020)	(0.025)
Finland	−0.697***	0.652***	−0.618***	0.618***			−0.316***	0.397***
	(0.042)	(0.029)	(0.031)	(0.034)			(0.011)	(0.013)
France	−0.064	0.050***	−0.109***	0.185***			0.255***	−0.148***
	(0.040)	(0.019)	(0.033)	(0.022)			(0.024)	(0.021)
United Kingdom	−0.205***	0.277***	−0.220***	0.380***			0.129***	0.077***
	(0.043)	(0.026)	(0.032)	(0.033)			(0.009)	(0.006)
Greece		−0.323***		−0.054				−0.452***
		(0.035)		(0.035)				(0.020)
Croatia		−0.131***		0.015		−0.151***		
		(0.025)		(0.023)		(0.040)		
Hungary	−0.052	0.030	0.104***	0.016			0.363***	−0.228***
	(0.039)	(0.018)	(0.029)	(0.021)			(0.025)	(0.027)
Ireland	−0.327***	0.141***	−0.331***	0.243***			0.001	−0.063***
	(0.040)	(0.023)	(0.030)	(0.030)			(0.014)	(0.008)
Israel	−0.079***	0.221***	−0.236***	0.443***			0.176***	0.060***
	(0.029)	(0.022)	(0.028)	(0.028)			(0.024)	(0.012)
Iceland	−0.181***	0.540***	−0.269***	0.517***			0.119***	0.276***
	(0.030)	(0.022)	(0.025)	(0.024)			(0.028)	(0.025)
Italy	−0.255***	0.072***	−0.225***	0.214***			0.076***	−0.144***
	(0.026)	(0.015)	(0.018)	(0.019)			(0.027)	(0.021)
Lithuania	−0.263***	−0.002	−0.368***	−0.040	−0.402***	−0.105***		
	(0.042)	(0.022)	(0.029)	(0.030)	(0.033)	(0.038)		
Latvia	−0.268***	−0.036	−0.182***	0.094***	−0.341***	−0.079		
	(0.058)	(0.031)	(0.048)	(0.035)	(0.029)	(0.048)		
Netherlands	−0.366***	0.357***	−0.350***	0.480***			−0.016	0.151***
	(0.045)	(0.019)	(0.036)	(0.023)			(0.025)	(0.020)
Norway	−0.492***	0.454***	−0.552***	0.555***			−0.168***	0.244***
	(0.042)	(0.023)	(0.034)	(0.025)			(0.024)	(0.022)
Poland	−0.127***	0.392***	0.096***	0.304***	−0.071**	0.288***		
	(0.034)	(0.018)	(0.031)	(0.019)	(0.036)	(0.033)		
Portugal	−0.388***	−0.067***	−0.220***	0.075***			0.059***	−0.268***
	(0.045)	(0.021)	(0.024)	(0.025)			(0.017)	(0.019)
Romania		−0.134***		0.082**		−0.148***		
		(0.030)		(0.032)		(0.053)		
Russian Federation	−0.017	0.004	−0.174***	0.050*			0.214***	−0.223***
	(0.041)	(0.021)	(0.038)	(0.027)			(0.040)	(0.033)
Sweden	−0.299***	0.336***	−0.128***	0.334***	−0.304***	0.276***		
	(0.045)	(0.028)	(0.036)	(0.035)	(0.031)	(0.028)		
Slovenia	−0.377***	0.288***	−0.528***	0.170***	−0.555***	0.181***		
	(0.033)	(0.017)	(0.029)	(0.020)	(0.035)	(0.032)		
Slovakia	−0.030	−0.040**	−0.295***	0.183***	−0.250***	−0.010		
	(0.038)	(0.019)	(0.031)	(0.019)	(0.020)	(0.026)		
Turkey		−0.741***		−0.425***				−0.847***
		(0.048)		(0.050)				(0.033)
Ukraine	−0.173***	−0.028	−0.082***	−0.110***	−0.171***	−0.199***		
	(0.040)	(0.021)	(0.029)	(0.027)	(0.034)	(0.043)		
Kosovo	−0.016	−0.286***	−0.121***	−0.129***			0.260***	−0.496***
	(0.020)	(0.010)	(0.017)	(0.013)			(0.041)	(0.031)
*Observations*	60,952	128,640	31,630	69,242	24,739	53,579	67,843	144,303
*Pseudo R-Squared*	0.0874	0.0624	0.144	0.0654	0.174	0.0843	0.180	0.0799

(1) the dependent variable is “Depression”, (2) the dependent variable is “Life Satisfaction” by considering the sample with persons with a good health. (3) the dependent variable is “Depression”, (4) the dependent variable is “Life Satisfaction” by considering the sample with persons with not a good health. (5) the dependent variable is “Depression”, (6) the dependent variable is “Life Satisfaction” by considering the sample with Eastern persons. (7) the dependent variable is “Depression”, (8) the dependent variable is “Life Satisfaction” by considering the sample with not Eastern persons. Sample survival indicates the marginal effects of the covariates on the survival across waves. Standard errors clustered at country level in parentheses.

*** $$p<0.01$$, ** $$p<0.05$$, * $$p<0.1$$

Country coefficients are those of the corresponding table 4, 5, 6. Albania is the omitted benchmark. Note that: (1) the dependent variable is “Depression”, (2) the dependent variable is “Life Satisfaction” by considering the sample with persons with a good health. (3) the dependent variable is “Depression”, (4) the dependent variable is “Life Satisfaction” by considering the sample with persons with not a good health. (5) the dependent variable is “Depression”, (6) the dependent variable is “Life Satisfaction” by considering the sample with Eastern persons. (7) the dependent variable is “Depression”, (8) the dependent variable is “Life Satisfaction” by considering the sample with not Eastern persons. Sample survival indicates the marginal effects of the covariates on the survival across waves. Standard errors clustered at country level in parentheses.

**Table 13:** Stability of the Gender paradox across Waves

Variables	Wave 3-6-7		Wave 3		Wave 6		Wave 7	
	(1)	(2)	(3)	(4)	(5)	(6)	(7)	(8)
	Depression	Life Sat.	Depression	Life Sat.	Depression	Life Sat.	Depression	Life Sat.
*Female*	0.178***	0.115***	0.181***	0.120***	0.185***	0.133***	0.173***	0.083***
	(0.030)	(0.010)	(0.049)	(0.016)	(0.029)	(0.015)	(0.040)	(0.014)
*Age class*								
0-19	0.140**	0.157***	0.213**	0.150***	0.151**	0.161***	0.096	0.168***
	(0.058)	(0.028)	(0.093)	(0.044)	(0.074)	(0.043)	(0.156)	(0.052)
20-29	0.071**	0.149***	0.032	0.155***	0.068	0.120***	0.136**	0.191***
	(0.029)	(0.016)	(0.049)	(0.026)	(0.046)	(0.020)	(0.065)	(0.025)
30-39	0.030	0.075***	0.017	0.044**	0.039	0.095***	0.048	0.084***
	(0.030)	(0.012)	(0.053)	(0.019)	(0.038)	(0.018)	(0.054)	(0.023)
50-59	−0.071***	0.024*	−0.026	0.002	−0.092***	0.018	−0.076*	0.057**
	(0.019)	(0.014)	(0.037)	(0.018)	(0.028)	(0.018)	(0.046)	(0.028)
60-69	−0.177***	0.108***	−0.218***	0.097***	−0.154***	0.105***	−0.154*	0.134***
	(0.032)	(0.025)	(0.042)	(0.028)	(0.054)	(0.030)	(0.083)	(0.039)
70–79	−0.242***	0.190***	−0.297***	0.169***	−0.198***	0.182***	−0.237**	0.226***
	(0.042)	(0.028)	(0.060)	(0.028)	(0.063)	(0.036)	(0.109)	(0.044)
80–89	−0.244***	0.224***	−0.282***	0.180***	−0.201***	0.266***	−0.249**	0.242***
	(0.041)	(0.037)	(0.072)	(0.051)	(0.064)	(0.053)	(0.100)	(0.055)
90+	−0.220**	0.255***	−0.047	0.073	−0.356*	0.348**	−0.213	0.332***
	(0.092)	(0.070)	(0.178)	(0.105)	(0.187)	(0.161)	(0.207)	(0.127)
*Employment relation*								
Student	−0.031	0.039**	−0.114*	0.067**	−0.102**	0.048*	0.105*	−0.006
	(0.032)	(0.019)	(0.069)	(0.029)	(0.049)	(0.028)	(0.064)	(0.026)
Unemployed, active	0.138***	−0.177***	0.109*	−0.176***	0.152***	−0.152***	0.118***	−0.196***
	(0.033)	(0.016)	(0.062)	(0.033)	(0.047)	(0.028)	(0.042)	(0.038)
Unemployed, not active	0.174***	−0.155***	0.150*	−0.141**	0.175***	−0.130***	0.174**	−0.195***
	(0.048)	(0.033)	(0.090)	(0.062)	(0.058)	(0.050)	(0.069)	(0.044)
Retired	−0.033	0.065***	−0.040	0.094***	−0.046	0.060***	−0.014	0.050**
	(0.031)	(0.016)	(0.051)	(0.025)	(0.050)	(0.019)	(0.063)	(0.024)
*Household’s income*								
2	−0.007	−0.035	0.068	−0.065	−0.010	−0.013	−0.032	−0.047
	(0.038)	(0.024)	(0.046)	(0.054)	(0.049)	(0.031)	(0.072)	(0.032)
3	−0.034	0.006	0.041	0.014	−0.059	0.055	−0.026	−0.051
	(0.037)	(0.026)	(0.061)	(0.062)	(0.052)	(0.034)	(0.058)	(0.032)
4	−0.038	−0.018	0.035	0.002	−0.035	−0.008	−0.062	−0.048*
	(0.030)	(0.021)	(0.074)	(0.064)	(0.053)	(0.036)	(0.049)	(0.028)
5	−0.057	0.011	0.006	0.016	−0.025	0.005	−0.094*	0.007
	(0.037)	(0.021)	(0.068)	(0.068)	(0.058)	(0.026)	(0.057)	(0.039)
6	−0.071*	0.015	−0.012	0.023	−0.030	0.051	−0.120*	−0.042
	(0.039)	(0.023)	(0.082)	(0.066)	(0.051)	(0.035)	(0.064)	(0.037)
7	−0.062	0.020	−0.015	0.014	−0.018	0.040	−0.097	−0.016
	(0.047)	(0.024)	(0.081)	(0.065)	(0.060)	(0.034)	(0.072)	(0.033)
8	−0.085**	0.015	−0.111	0.005	−0.048	0.020	−0.046	−0.007
	(0.040)	(0.022)	(0.087)	(0.068)	(0.059)	(0.032)	(0.072)	(0.038)
9	−0.087*	0.029	−0.024	−0.005	0.011	0.049	−0.234***	0.022
	(0.047)	(0.030)	(0.089)	(0.073)	(0.074)	(0.038)	(0.070)	(0.038)
10	−0.200***	0.041	−0.215*	−0.039	−0.161*	0.096***	−0.178*	0.013
	(0.064)	(0.026)	(0.118)	(0.076)	(0.093)	(0.031)	(0.098)	(0.048)
*Household size*	−0.013	0.044***	−0.032**	0.049***	−0.011	0.033***	−0.003	0.057***
	(0.009)	(0.006)	(0.015)	(0.009)	(0.011)	(0.006)	(0.019)	(0.008)
*Education status*								
No or unfinished	0.039	0.089*	−0.251***	0.175***				
	(0.055)	(0.054)	(0.080)	(0.056)				
Primary	0.203***	0.121***	0.170*	0.066	0.264***	0.148***	0.169*	0.098***
	(0.037)	(0.031)	(0.097)	(0.086)	(0.055)	(0.044)	(0.092)	(0.036)
Lower Secondary	0.154***	0.022	0.138	0.002	0.209***	0.027	0.102	0.025
	(0.035)	(0.029)	(0.087)	(0.056)	(0.042)	(0.035)	(0.068)	(0.037)
Upper Secondary	0.044	0.035	0.013	−0.023	0.101**	0.081**	0.007	0.025
	(0.028)	(0.024)	(0.090)	(0.055)	(0.044)	(0.036)	(0.054)	(0.027)
Post-Secondary	0.074**	−0.003	0.045	−0.045	0.119***	0.026	0.044	−0.011
	(0.030)	(0.021)	(0.071)	(0.048)	(0.039)	(0.024)	(0.070)	(0.029)
First Level Tertiary	0.029	0.037*	−0.008	0.024	0.077*	0.030	−0.012	0.070***
	(0.029)	(0.019)	(0.084)	(0.045)	(0.044)	(0.022)	(0.058)	(0.024)
Second Level Tertiary	0.026	0.019	0.029	−0.048	0.061	0.040*	−0.043	0.040*
	(0.040)	(0.014)	(0.098)	(0.033)	(0.054)	(0.022)	(0.056)	(0.021)
*Marital Status*								
Registered partner	0.095	−0.232***	0.111	−0.254***	−0.411	−0.101	0.174	−0.252**
	(0.067)	(0.043)	(0.074)	(0.044)	(0.322)	(0.084)	(0.242)	(0.106)
Separated	0.314***	−0.451***			0.266**	−0.404***	0.371***	−0.481***
	(0.067)	(0.043)			(0.121)	(0.066)	(0.112)	(0.103)
Divorced	0.110***	−0.254***	0.028	−0.242***	0.166***	−0.280***	0.110**	−0.242***
	(0.025)	(0.017)	(0.066)	(0.032)	(0.034)	(0.023)	(0.047)	(0.025)
Widowed	0.175***	−0.343***	0.214***	−0.323***	0.158***	−0.383***	0.181***	−0.317***
	(0.028)	(0.026)	(0.047)	(0.042)	(0.039)	(0.033)	(0.059)	(0.025)
Never Married	0.037*	−0.295***	0.049	−0.307***	−0.012	−0.303***	0.098**	−0.282***
	(0.019)	(0.018)	(0.056)	(0.031)	(0.030)	(0.020)	(0.046)	(0.026)
*Self health*								
Good	0.206***	−0.309***	0.171***	−0.311***	0.188***	−0.278***	0.275***	−0.357***
	(0.030)	(0.016)	(0.038)	(0.021)	(0.048)	(0.022)	(0.064)	(0.020)
Fair	0.579***	−0.580***	0.560***	−0.591***	0.509***	−0.542***	0.714***	−0.639***
	(0.033)	(0.025)	(0.053)	(0.033)	(0.045)	(0.028)	(0.069)	(0.034)
Bad	1.101***	−0.897***	1.056***	−0.918***	1.046***	−0.830***	1.260***	−0.991***
	(0.043)	(0.036)	(0.060)	(0.049)	(0.049)	(0.043)	(0.098)	(0.054)
Very Bad	1.584***	−1.276***	1.619***	−1.296***	1.509***	−1.227***	1.719***	−1.339***
	(0.070)	(0.042)	(0.084)	(0.075)	(0.076)	(0.055)	(0.154)	(0.082)
*Social meeting*								
Less than once a month	−0.291***	0.181***	−0.359***	0.217***	−0.246***	0.163**	−0.322***	0.198***
	(0.056)	(0.044)	(0.076)	(0.083)	(0.085)	(0.066)	(0.080)	(0.066)
Once a month	−0.414***	0.353***	−0.480***	0.354***	−0.389***	0.347***	−0.403***	0.370***
	(0.061)	(0.043)	(0.113)	(0.073)	(0.081)	(0.062)	(0.097)	(0.065)
Several times a month	−0.568***	0.442***	−0.619***	0.486***	−0.560***	0.410***	−0.538***	0.463***
	(0.050)	(0.041)	(0.089)	(0.064)	(0.075)	(0.064)	(0.075)	(0.060)
Once a week	−0.501***	0.483***	−0.555***	0.508***	−0.507***	0.475***	−0.450***	0.494***
	(0.052)	(0.045)	(0.086)	(0.066)	(0.077)	(0.070)	(0.076)	(0.061)
Several times a week	−0.572***	0.586***	−0.647***	0.622***	−0.552***	0.544***	−0.533***	0.628***
	(0.049)	(0.046)	(0.085)	(0.062)	(0.075)	(0.070)	(0.063)	(0.061)
Every day	−0.525***	0.715***	−0.555***	0.749***	−0.518***	0.684***	−0.519***	0.744***
	(0.048)	(0.047)	(0.082)	(0.070)	(0.083)	(0.073)	(0.060)	(0.062)
*Placement on left right scale*								
1	−0.098**	−0.131***	0.017	−0.174***	−0.140*	−0.070	−0.122	−0.173***
	(0.039)	(0.040)	(0.099)	(0.067)	(0.080)	(0.051)	(0.090)	(0.065)
2	−0.077*	−0.167***	−0.120	−0.250***	−0.117*	−0.105**	0.029	−0.176***
	(0.043)	(0.042)	(0.106)	(0.074)	(0.060)	(0.047)	(0.073)	(0.061)
3	−0.077**	−0.170***	−0.128	−0.238***	−0.080*	−0.138***	−0.001	−0.155**
	(0.033)	(0.043)	(0.080)	(0.071)	(0.047)	(0.053)	(0.051)	(0.068)
4	−0.049	−0.191***	−0.079	−0.238***	−0.011	−0.167***	−0.055	−0.179***
	(0.041)	(0.034)	(0.091)	(0.066)	(0.045)	(0.039)	(0.070)	(0.062)
5	−0.116***	−0.083**	−0.101	−0.159**	−0.121***	−0.032	−0.117**	−0.087
	(0.029)	(0.039)	(0.083)	(0.066)	(0.029)	(0.045)	(0.054)	(0.058)
6	−0.099***	−0.116***	−0.106	−0.190***	−0.085**	−0.044	−0.100	−0.145**
	(0.036)	(0.041)	(0.089)	(0.061)	(0.038)	(0.050)	(0.063)	(0.063)
7	−0.136***	−0.102**	−0.185**	−0.195***	−0.134***	−0.018	−0.069	−0.130**
	(0.038)	(0.041)	(0.081)	(0.065)	(0.045)	(0.053)	(0.068)	(0.054)
8	−0.049	0.004	−0.053	−0.051	−0.096**	0.063	0.036	−0.025
	(0.034)	(0.040)	(0.078)	(0.066)	(0.047)	(0.052)	(0.071)	(0.047)
9	−0.116**	0.090**	−0.149	−0.099	−0.165*	0.214***	0.003	0.068
	(0.057)	(0.039)	(0.091)	(0.068)	(0.089)	(0.049)	(0.088)	(0.064)
10	−0.049	0.243***	0.179*	0.102	−0.130**	0.280***	−0.103	0.303***
	(0.051)	(0.052)	(0.100)	(0.076)	(0.055)	(0.059)	(0.081)	(0.088)
*Feeling about Household’s income nowadays*								
Copying on present income	0.097***	−0.255***	0.114***	−0.258***	0.128***	−0.235***	0.054	−0.275***
	(0.022)	(0.017)	(0.039)	(0.018)	(0.039)	(0.018)	(0.034)	(0.025)
Difficult on present income	0.334***	−0.552***	0.329***	−0.574***	0.376***	−0.533***	0.293***	−0.544***
	(0.031)	(0.038)	(0.048)	(0.039)	(0.055)	(0.041)	(0.053)	(0.048)
Very difficult on present income	0.627***	−0.893***	0.633***	−0.880***	0.673***	−0.895***	0.558***	−0.850***
	(0.040)	(0.043)	(0.064)	(0.062)	(0.062)	(0.042)	(0.069)	(0.072)
*Waves*								
6	−0.090**	0.100***						
	(0.038)	(0.033)						
7	−0.138***	0.091***						
	(0.037)	(0.032)						
*Constant*	−1.314***		−1.288***		−1.445***		−1.884***	
	(0.062)		(0.156)		(0.095)		(0.130)	
*Observations*	92,582	92,542	26,030	26,040	37,969	37,910	28,583	28,592
*Pseudo R-Squared*	0.178	0.0818	0.175	0.0820	0.183	0.0885	0.179	0.0739
Country effects across Waves								
*Country*								
Austria	−0.327***	0.183***						
	(0.031)	(0.035)						
Belgium	−0.119***	0.382***	0.010	0.420***	−0.175***	0.337***	0.194***	0.137***
	(0.030)	(0.022)	(0.035)	(0.033)	(0.031)	(0.024)	(0.015)	(0.011)
Bulgaria	−0.297***	−0.397***	0.099	−0.538***	−0.351***	−0.417***		
	(0.023)	(0.026)	(0.066)	(0.044)	(0.029)	(0.025)		
Switzerland	−0.218***	0.532***	−0.062	0.627***	−0.310***	0.456***	0.090***	0.280***
	(0.034)	(0.026)	(0.042)	(0.038)	(0.036)	(0.029)	(0.011)	(0.015)
Cyprus	−0.252***	0.330***	−0.031**	0.323***	−0.196***	0.198***		
	(0.022)	(0.030)	(0.015)	(0.015)	(0.021)	(0.017)		
Czech Republic	0.177***	−0.039*			0.181***	−0.161***	0.456***	−0.189***
	(0.027)	(0.022)			(0.037)	(0.021)	(0.019)	(0.015)
Germany	−0.181***	0.365***	−0.163***	0.230***	−0.189***	0.415***	0.164***	0.160***
	(0.031)	(0.022)	(0.030)	(0.021)	(0.034)	(0.024)	(0.013)	(0.014)
Denmark	−0.317***	0.648***	−0.263***	0.718***	−0.430***	0.665***	0.096***	0.316***
	(0.031)	(0.031)	(0.033)	(0.043)	(0.036)	(0.034)	(0.015)	(0.020)
Estonia	−0.265***	0.238***			−0.290***	0.203***		
	(0.021)	(0.019)			(0.034)	(0.022)		
Spain	−0.173***	0.419***	−0.312***	0.486***	−0.067***	0.426***	0.126***	0.097***
	(0.027)	(0.020)	(0.045)	(0.036)	(0.023)	(0.021)	(0.024)	(0.015)
Finland	−0.646***	0.644***	−0.328***	0.508***	−0.646***	0.620***	−0.342***	0.430***
	(0.027)	(0.033)	(0.015)	(0.012)	(0.031)	(0.030)	(0.018)	(0.015)
France	−0.079***	0.098***	0.084**	0.116***	−0.131***	0.063***	0.159***	−0.147***
	(0.029)	(0.021)	(0.033)	(0.027)	(0.027)	(0.021)	(0.014)	(0.012)
United Kingdom	−0.198***	0.308***	0.163***	0.158***	−0.193***	0.296***	0.075***	0.093***
	(0.028)	(0.031)	(0.015)	(0.007)	(0.029)	(0.024)	(0.015)	(0.010)
Hungary	0.039	−0.039*			0.051*	−0.083***	0.288***	−0.250***
	(0.024)	(0.021)			(0.030)	(0.018)	(0.027)	(0.026)
Ireland	−0.317***	0.165***	−0.076***	0.211***	−0.328***	0.051***	0.059***	−0.077***
	(0.026)	(0.025)	(0.019)	(0.009)	(0.031)	(0.018)	(0.013)	(0.013)
Israel	−0.148***	0.326***			−0.145***	0.363***	0.146***	0.009
	(0.020)	(0.020)			(0.024)	(0.021)	(0.029)	(0.017)
Iceland	−0.192***	0.635***			−0.196***	0.611***		
	(0.018)	(0.026)			(0.031)	(0.029)		
Italy	−0.239***	0.111***			−0.238***	0.091***		
	(0.016)	(0.015)			(0.018)	(0.016)		
Lithuania	−0.336***	−0.067***			−0.374***	−0.089***	−0.003	−0.311***
	(0.027)	(0.023)			(0.034)	(0.022)	(0.031)	(0.024)
Latvia	−0.214***	−0.084**	−0.138***	−0.094***				
	(0.046)	(0.038)	(0.017)	(0.009)				
Netherlands	−0.348***	0.388***	−0.290***	0.407***	−0.399***	0.356***	0.013	0.151***
	(0.032)	(0.024)	(0.035)	(0.032)	(0.032)	(0.026)	(0.016)	(0.013)
Norway	−0.503***	0.472***	−0.432***	0.483***	−0.445***	0.497***	−0.273***	0.190***
	(0.029)	(0.027)	(0.039)	(0.041)	(0.033)	(0.031)	(0.017)	(0.017)
Poland	0.005	0.320***	0.090***	0.292***	−0.039	0.303***	0.326***	0.093***
	(0.029)	(0.019)	(0.014)	(0.011)	(0.026)	(0.021)	(0.028)	(0.018)
Portugal	−0.258***	−0.083***	0.131***	−0.358***	−0.313***	−0.210***	0.027	−0.112***
	(0.025)	(0.028)	(0.035)	(0.026)	(0.032)	(0.023)	(0.034)	(0.021)
Russian Federation	−0.129***	0.011	−0.030	0.010	−0.168***	−0.031		
	(0.032)	(0.024)	(0.027)	(0.025)	(0.041)	(0.023)		
Sweden	−0.216***	0.340***	0.168***	0.285***	−0.249***	0.279***	0.056***	0.077***
	(0.030)	(0.032)	(0.018)	(0.013)	(0.035)	(0.028)	(0.018)	(0.018)
Slovenia	−0.463***	0.189***	−0.405***	0.266***	−0.628***	0.155***	−0.044*	−0.143***
	(0.026)	(0.020)	(0.025)	(0.023)	(0.024)	(0.022)	(0.026)	(0.013)
Slovakia	−0.173***	0.031			−0.299***	0.016		
	(0.030)	(0.021)			(0.027)	(0.021)		
Ukraine	−0.107***	−0.054***			−0.119***	−0.064***		
	(0.024)	(0.018)			(0.036)	(0.020)		
Kosovo	−0.054***	−0.230***			−0.057***	−0.229***		
	(0.016)	(0.011)			(0.019)	(0.014)		
*Observations*	92,582	92,542	26,030	26,040	37,969	37,910	28,583	28,592
*Pseudo R-Squared*	0.178	0.0818	0.175	0.0820	0.183	0.0885	0.179	0.0739

(1) the dependent variable is “Depression”, (2) the dependent variable is “Life Satisfaction” by considering the sample with all waves. (3) the dependent variable is “Depression”, (4) the dependent variable is “Life Satisfaction” by considering the sample with persons with the third wave. (5) the dependent variable is “Depression”, (6) the dependent variable is “Life Satisfaction” by considering the sample with the sixth wave. (7) the dependent variable is “Depression”, (8) the dependent variable is “Life Satisfaction” by considering the sample with seventh wave. Sample survival indicates the marginal effects of the covariates on the survival across waves. Standard errors clustered at country level in parentheses.

*** $$p<0.01$$, ** $$p<0.05$$, * $$p<0.1$$

Country coefficients are those of the corresponding table 7. Albania is the omitted benchmark. Note that: (1) the dependent variable is “Depression”, (2) the dependent variable is “Life Satisfaction” by considering the sample with all waves. (3) the dependent variable is “Depression”, (4) the dependent variable is “Life Satisfaction” by considering the sample with persons with the third wave. (5) the dependent variable is “Depression”, (6) the dependent variable is “Life Satisfaction” by considering the sample with the sixth wave. (7) the dependent variable is “Depression”, (8) the dependent variable is “Life Satisfaction” by considering the sample with seventh wave. Sample survival indicates the marginal effects of the covariates on the survival across waves. Standard errors clustered at country level in parentheses.

**Table 14:** Stability of the Gender paradox across different seasons

Variables	Summer		Autumn		Winter		Spring	
	(1)	(2)	(3)	(4)	(5)	(6)	(7)	(8)
	Depression	Life Sat.	Depression	Life Sat.	Depression	Life Sat.	Depression	Life Sat.
*Female*	0.151**	0.112***	0.178***	0.114***	0.180***	0.119***	0.203***	0.113***
	(0.065)	(0.015)	(0.033)	(0.014)	(0.039)	(0.011)	(0.067)	(0.028)
*Age class*								
0–19	0.340	0.154*	0.153**	0.148***	0.114	0.103**	−0.071	0.177***
	(0.234)	(0.081)	(0.077)	(0.029)	(0.099)	(0.041)	(0.257)	(0.047)
20–29	0.141*	0.139***	0.116**	0.141***	−0.004	0.116***	0.026	0.147***
	(0.076)	(0.036)	(0.053)	(0.018)	(0.044)	(0.027)	(0.123)	(0.040)
30–39	−0.021	0.103***	0.060*	0.081***	−0.004	0.071***	0.008	0.067**
	(0.147)	(0.030)	(0.036)	(0.014)	(0.064)	(0.015)	(0.058)	(0.031)
50–59	−0.155	0.021	−0.072**	0.037***	−0.065	0.023	0.018	0.009
	(0.118)	(0.035)	(0.035)	(0.014)	(0.040)	(0.020)	(0.065)	(0.031)
60–69	−0.333**	0.073	−0.165***	0.146***	−0.184***	0.107***	−0.039	0.070*
	(0.137)	(0.063)	(0.035)	(0.031)	(0.052)	(0.030)	(0.114)	(0.041)
70–79	−0.395***	0.152**	−0.199***	0.247***	−0.276***	0.136***	−0.142	0.138***
	(0.093)	(0.067)	(0.056)	(0.029)	(0.076)	(0.042)	(0.143)	(0.047)
80–89	−0.430***	0.197***	−0.180***	0.302***	−0.357***	0.201***	−0.038	0.167**
	(0.124)	(0.073)	(0.060)	(0.034)	(0.088)	(0.062)	(0.192)	(0.073)
90+	−0.126	0.202	−0.074	0.348***	−0.559***	0.158*	−0.255	0.174**
	(0.340)	(0.175)	(0.141)	(0.046)	(0.203)	(0.094)	(0.360)	(0.088)
*Employment relation*								
Student	−0.230***	0.052	0.008	0.030**	−0.045	0.066***	−0.117	0.026
	(0.081)	(0.041)	(0.056)	(0.014)	(0.042)	(0.024)	(0.177)	(0.040)
Unemployed, active	0.272**	−0.182***	0.112***	−0.200***	0.110**	−0.159***	0.220**	−0.144***
	(0.107)	(0.047)	(0.041)	(0.029)	(0.052)	(0.028)	(0.111)	(0.031)
Unemployed, not active	0.234**	−0.148**	0.174***	−0.154***	0.162**	−0.114***	0.138	−0.163*
	(0.115)	(0.063)	(0.065)	(0.026)	(0.065)	(0.042)	(0.114)	(0.086)
Retired	0.183***	0.042	−0.067	0.049***	−0.057	0.090***	0.037	0.036
	(0.070)	(0.034)	(0.042)	(0.016)	(0.062)	(0.027)	(0.048)	(0.028)
*Household’s income*								
2	−0.074	0.047	0.003	0.003	−0.017	−0.005	0.036	0.090**
	(0.078)	(0.072)	(0.039)	(0.017)	(0.062)	(0.021)	(0.102)	(0.036)
3	0.006	0.017	−0.025	0.007	−0.102*	0.050*	0.066	0.038
	(0.058)	(0.070)	(0.054)	(0.018)	(0.058)	(0.030)	(0.091)	(0.058)
4	−0.143*	0.065	−0.040	0.006	−0.024	0.022	−0.014	0.009
	(0.074)	(0.063)	(0.045)	(0.024)	(0.046)	(0.030)	(0.095)	(0.041)
5	−0.195*	0.103	0.028	0.026	−0.167***	0.018	0.033	0.036
	(0.118)	(0.076)	(0.041)	(0.025)	(0.048)	(0.029)	(0.142)	(0.041)
6	−0.195***	0.189**	−0.021	0.033	−0.104*	0.033	−0.078	0.012
	(0.066)	(0.074)	(0.046)	(0.022)	(0.059)	(0.032)	(0.119)	(0.049)
7	−0.221**	0.110	−0.014	0.052*	−0.119*	0.073**	0.038	0.070
	(0.093)	(0.090)	(0.059)	(0.027)	(0.072)	(0.031)	(0.111)	(0.051)
8	−0.001	0.138	−0.072	0.052**	−0.138**	0.044	−0.001	0.086*
	(0.080)	(0.094)	(0.049)	(0.026)	(0.063)	(0.036)	(0.088)	(0.044)
9	−0.063	0.162*	−0.037	0.058*	−0.181**	0.074**	−0.001	0.076
	(0.090)	(0.096)	(0.066)	(0.031)	(0.082)	(0.031)	(0.129)	(0.049)
10	−0.260**	0.208*	−0.092	0.050*	−0.346***	0.115***	−0.225	0.105*
	(0.131)	(0.109)	(0.068)	(0.028)	(0.082)	(0.038)	(0.225)	(0.054)
*Household size*	−0.020	0.035***	−0.022**	0.050***	−0.005	0.037***	0.005	0.046***
	(0.032)	(0.007)	(0.010)	(0.007)	(0.014)	(0.005)	(0.024)	(0.010)
*Education status*								
No or unfinished	0.342**	0.034	−0.009	0.115**	0.129	0.073	−0.239	0.109
	(0.143)	(0.069)	(0.062)	(0.045)	(0.099)	(0.053)	(0.192)	(0.095)
Primary	0.298**	−0.093*	0.177***	0.083**	0.217***	0.132***	0.143	0.088**
	(0.120)	(0.048)	(0.057)	(0.036)	(0.058)	(0.039)	(0.116)	(0.039)
Lower Secondary	0.227*	−0.102*	0.185***	0.031	0.130**	0.016	0.021	−0.014
	(0.129)	(0.057)	(0.047)	(0.032)	(0.051)	(0.035)	(0.067)	(0.045)
Upper Secondary	0.185**	−0.118***	0.017	0.050*	0.056	0.025	0.074	−0.041
	(0.088)	(0.042)	(0.046)	(0.027)	(0.049)	(0.022)	(0.072)	(0.035)
Post-Secondary	0.120	−0.070**	0.070*	0.019	0.100*	−0.011	−0.020	−0.046
	(0.085)	(0.031)	(0.041)	(0.026)	(0.054)	(0.018)	(0.070)	(0.032)
First Level Tertiary	0.143	−0.073***	0.052	0.054***	−0.035	0.039*	0.055	−0.022
	(0.096)	(0.028)	(0.050)	(0.020)	(0.035)	(0.023)	(0.145)	(0.029)
Second Level Tertiary	0.135*	0.004	0.076	0.013	−0.060	−0.001	−0.056	−0.012
	(0.075)	(0.054)	(0.047)	(0.014)	(0.069)	(0.016)	(0.100)	(0.033)
*Marital Status*								
Registered partner	−0.149	−0.196*	0.110	−0.139***	0.105	−0.197***	−0.243	−0.245***
	(0.146)	(0.116)	(0.090)	(0.045)	(0.106)	(0.049)	(0.390)	(0.086)
Separated	0.034	−0.434**	0.419***	−0.517***	0.259*	−0.503***	0.180	−0.154
	(0.457)	(0.203)	(0.121)	(0.042)	(0.149)	(0.046)	(0.290)	(0.126)
Divorced	0.071	−0.267***	0.113***	−0.262***	0.081*	−0.231***	0.246***	−0.281***
	(0.068)	(0.045)	(0.036)	(0.020)	(0.048)	(0.019)	(0.069)	(0.033)
Widowed	−0.034	−0.206***	0.238***	−0.354***	0.168***	−0.309***	0.153**	−0.267***
	(0.105)	(0.042)	(0.038)	(0.033)	(0.046)	(0.041)	(0.063)	(0.038)
Never Married	−0.085	−0.235***	0.035	−0.307***	0.047	−0.243***	0.132**	−0.228***
	(0.096)	(0.034)	(0.025)	(0.019)	(0.035)	(0.023)	(0.065)	(0.031)
*Self health*								
Good	0.201	−0.264***	0.197***	−0.335***	0.220***	−0.314***	0.170**	−0.277***
	(0.140)	(0.044)	(0.040)	(0.018)	(0.061)	(0.024)	(0.076)	(0.035)
Fair	0.619***	−0.584***	0.599***	−0.633***	0.563***	−0.570***	0.512***	−0.532***
	(0.195)	(0.064)	(0.036)	(0.025)	(0.060)	(0.034)	(0.077)	(0.045)
Bad	1.115***	−0.924***	1.136***	−0.960***	1.095***	−0.874***	1.051***	−0.879***
	(0.165)	(0.098)	(0.036)	(0.037)	(0.108)	(0.047)	(0.084)	(0.057)
Very Bad	1.591***	−1.201***	1.610***	−1.266***	1.580***	−1.204***	1.544***	−1.364***
	(0.206)	(0.084)	(0.083)	(0.046)	(0.101)	(0.064)	(0.160)	(0.122)
*Social meeting*								
Less than once a month	−0.476***	0.460***	−0.239***	0.196***	−0.234***	0.123***	−0.504***	0.181**
	(0.158)	(0.070)	(0.072)	(0.054)	(0.080)	(0.048)	(0.089)	(0.080)
Once a month	−0.552***	0.643***	−0.396***	0.333***	−0.351***	0.305***	−0.594***	0.345***
	(0.158)	(0.087)	(0.081)	(0.050)	(0.085)	(0.059)	(0.161)	(0.079)
Several times a month	−0.699***	0.698***	−0.535***	0.464***	−0.544***	0.383***	−0.679***	0.412***
	(0.161)	(0.074)	(0.067)	(0.048)	(0.080)	(0.054)	(0.132)	(0.070)
Once a week	−0.596***	0.727***	−0.425***	0.485***	−0.517***	0.422***	−0.706***	0.476***
	(0.122)	(0.083)	(0.073)	(0.051)	(0.076)	(0.056)	(0.169)	(0.078)
Several times a week	−0.688***	0.806***	−0.502***	0.621***	−0.572***	0.534***	−0.803***	0.573***
	(0.147)	(0.088)	(0.067)	(0.047)	(0.071)	(0.055)	(0.136)	(0.076)
Every day	−0.676***	0.906***	−0.477***	0.747***	−0.487***	0.672***	−0.784***	0.709***
	(0.131)	(0.103)	(0.063)	(0.046)	(0.075)	(0.057)	(0.188)	(0.080)
*Placement on left right scale*								
1	−0.261	−0.149	−0.135*	−0.115***	−0.047	−0.087**	−0.027	−0.041
	(0.169)	(0.096)	(0.070)	(0.035)	(0.074)	(0.039)	(0.137)	(0.057)
2	−0.119	−0.089	−0.056	−0.218***	−0.134**	−0.115***	0.062	−0.058
	(0.255)	(0.095)	(0.057)	(0.030)	(0.061)	(0.042)	(0.091)	(0.052)
3	−0.072	−0.174	−0.057	−0.246***	−0.142**	−0.098**	0.065	−0.098*
	(0.218)	(0.118)	(0.042)	(0.030)	(0.061)	(0.039)	(0.077)	(0.050)
4	−0.153	−0.121	0.024	−0.253***	−0.167***	−0.142***	0.045	−0.121**
	(0.157)	(0.082)	(0.055)	(0.028)	(0.051)	(0.032)	(0.090)	(0.059)
5	−0.093	−0.075	−0.106**	−0.141***	−0.149***	−0.020	−0.053	−0.010
	(0.129)	(0.097)	(0.043)	(0.027)	(0.038)	(0.044)	(0.073)	(0.048)
6	−0.139	−0.067	−0.078*	−0.201***	−0.143**	−0.066	0.004	−0.043
	(0.162)	(0.105)	(0.040)	(0.030)	(0.061)	(0.044)	(0.070)	(0.070)
7	−0.099	−0.085	−0.179***	−0.174***	−0.142**	−0.038	0.070	−0.059
	(0.135)	(0.083)	(0.044)	(0.028)	(0.066)	(0.047)	(0.088)	(0.060)
8	−0.086	0.067	−0.051	−0.064**	−0.073	0.092*	0.122	0.053
	(0.156)	(0.103)	(0.049)	(0.030)	(0.056)	(0.054)	(0.127)	(0.066)
9	−0.387**	0.153	−0.026	0.003	−0.199	0.181***	0.029	0.162**
	(0.163)	(0.111)	(0.077)	(0.035)	(0.125)	(0.042)	(0.112)	(0.072)
10	−0.225	0.289***	−0.042	0.177***	−0.047	0.263***	0.014	0.314***
	(0.177)	(0.107)	(0.053)	(0.048)	(0.089)	(0.060)	(0.110)	(0.082)
*Feeling about Household’s income nowadays*								
Copying on present income	0.074	−0.160***	0.106***	−0.252***	0.072	−0.231***	0.162**	−0.214***
	(0.097)	(0.035)	(0.025)	(0.018)	(0.053)	(0.021)	(0.073)	(0.031)
Difficult on present income	0.294*	−0.353***	0.351***	−0.555***	0.309***	−0.500***	0.390***	−0.497***
	(0.166)	(0.045)	(0.032)	(0.036)	(0.056)	(0.032)	(0.086)	(0.051)
Very difficult on present income	0.646***	−0.587***	0.632***	−0.843***	0.608***	−0.803***	0.646***	−0.810***
	(0.143)	(0.062)	(0.055)	(0.044)	(0.068)	(0.044)	(0.082)	(0.049)
*Waves*								
4		0.333***		0.033*		−0.001		−0.141**
		(0.028)		(0.019)		(0.035)		(0.066)
5		0.251***		0.048*		0.069*		0.037
		(0.060)		(0.026)		(0.040)		(0.072)
6	0.543***	0.262***	−0.114***	0.127***	−0.065	0.089**	−0.300**	0.009
	(0.070)	(0.052)	(0.023)	(0.033)	(0.067)	(0.038)	(0.137)	(0.065)
7	0.528***	−0.008	−0.152***	0.105***	−0.084	0.101*	−0.366**	0.058
	(0.061)	(0.067)	(0.036)	(0.027)	(0.057)	(0.053)	(0.172)	(0.074)
8		0.370***		0.161***		0.209***		0.194***
		(0.105)		(0.034)		(0.045)		(0.075)
*Constant*	−1.834***		−1.662***		−1.294***		−1.646***	
	(0.321)		(0.087)		(0.115)		(0.273)	
*Observations*	6,514	13,962	49,935	102,828	27,893	61,591	8,187	18,158
*Pseudo R-Squared*	0.190	0.0793	0.178	0.0760	0.176	0.0798	0.185	0.0856
Country effects across different seasons								
*Country*								
Austria					−0.222***	0.093*		
					(0.055)	(0.056)		
Belgium			0.112***	0.148***	−0.005	0.359***		0.102***
			(0.037)	(0.015)	(0.061)	(0.031)		(0.030)
Bulgaria			−0.312***	−0.762***	−0.339***	−0.402***	0.227*	−0.653***
			(0.051)	(0.040)	(0.040)	(0.039)	(0.131)	(0.082)
Switzerland	0.716***	0.518***	−0.045	0.298***	0.087	0.459***	0.346**	0.317***
	(0.086)	(0.068)	(0.039)	(0.017)	(0.057)	(0.031)	(0.144)	(0.042)
Cyprus		0.057*	0.042**	0.082***	−0.278***	0.260***		0.004
		(0.031)	(0.020)	(0.014)	(0.029)	(0.026)		(0.037)
Czech Republic		−0.345***	0.161***	−0.280***	0.205***	−0.032	0.876***	−0.313***
		(0.072)	(0.052)	(0.019)	(0.045)	(0.031)	(0.126)	(0.051)
Germany	−0.656***	0.240***	0.064	0.148***	−0.101*	0.320***		0.080*
	(0.082)	(0.078)	(0.044)	(0.018)	(0.060)	(0.027)		(0.045)
Denmark			−0.067	0.446***	−0.274***	0.625***	0.160	0.407***
			(0.041)	(0.024)	(0.046)	(0.031)	(0.115)	(0.066)
Estonia			−0.060	0.046**	0.071*	0.116***		−0.309***
			(0.047)	(0.019)	(0.041)	(0.031)		(0.055)
Spain		0.054***	−0.259***	0.202***	−0.122***	0.383***	0.505***	0.065*
		(0.016)	(0.044)	(0.032)	(0.043)	(0.022)	(0.079)	(0.036)
Finland			−0.418***	0.400***	−0.500***	0.595***		−0.210***
			(0.025)	(0.013)	(0.039)	(0.045)		(0.068)
France	0.302***	−0.188**	0.155***	−0.151***	−0.001	0.040	0.301***	−0.124*
	(0.087)	(0.077)	(0.040)	(0.023)	(0.049)	(0.029)	(0.112)	(0.072)
United Kingdom		−0.544***	0.048**	0.079***	−0.106***	0.207***		0.104*
		(0.053)	(0.019)	(0.008)	(0.038)	(0.034)		(0.054)
Greece		−0.688***		−0.457***				−0.602***
		(0.031)		(0.029)				(0.040)
Croatia				−0.335***		−0.052		−7.024***
				(0.027)		(0.038)		(0.254)
Hungary	−0.194***	−0.325***	0.360***	−0.106***	0.148***	−0.106***	0.404***	−0.242***
	(0.067)	(0.119)	(0.050)	(0.027)	(0.031)	(0.020)	(0.065)	(0.033)
Ireland	−0.047	0.355***	0.027	−0.067***	−0.329***	0.133***		−0.209***
	(0.037)	(0.037)	(0.024)	(0.011)	(0.038)	(0.032)		(0.040)
Israel	−0.249***	0.198**	0.193***	0.102***	−0.071**	0.240***	0.343***	−0.068**
	(0.096)	(0.083)	(0.038)	(0.015)	(0.032)	(0.040)	(0.082)	(0.030)
Iceland		−0.010	−0.050	0.409***	0.016	0.442***		−0.033
		(0.139)	(0.045)	(0.027)	(0.036)	(0.038)		(0.034)
Italy	−0.129***	−0.196**	0.026	−0.109***		−0.443***		
	(0.039)	(0.078)	(0.039)	(0.018)		(0.036)		
Lithuania	−0.210***	−0.391***		−0.318***		−0.008	0.123**	−0.369***
	(0.042)	(0.032)		(0.014)		(0.053)	(0.051)	(0.015)
Latvia	0.531***	−0.270***		0.792***				−0.215***
	(0.097)	(0.063)		(0.033)				(0.061)
Netherlands		−0.034	−0.138***	0.160***	−0.226***	0.303***	0.000	0.187***
		(0.079)	(0.040)	(0.017)	(0.052)	(0.028)	(0.127)	(0.036)
Norway	−0.274***	0.190***	−0.296***	0.255***	−0.279***	0.394***		
	(0.065)	(0.071)	(0.041)	(0.020)	(0.044)	(0.030)		
Poland	0.102**	0.282***	0.257***	0.135***	0.352***	0.260***	0.494***	−0.016
	(0.051)	(0.078)	(0.051)	(0.022)	(0.044)	(0.032)	(0.049)	(0.017)
Portugal	−0.079	−0.084	−0.012	−0.191***	−0.255***	−0.143***	0.286***	−0.256***
	(0.070)	(0.072)	(0.026)	(0.020)	(0.038)	(0.035)	(0.068)	(0.044)
Romania						−0.056		
						(0.038)		
Russian Federation			0.075	−0.196***	0.210***	−0.069**		−0.367***
			(0.049)	(0.032)	(0.068)	(0.032)		(0.045)
Sweden	−0.396***	0.365***	0.033	0.102***	−0.085*	0.277***	−0.033	0.010
	(0.095)	(0.074)	(0.022)	(0.012)	(0.049)	(0.039)	(0.117)	(0.070)
Slovenia			−0.211***	−0.017	−0.438***	0.210***		
			(0.044)	(0.020)	(0.037)	(0.030)		
Slovakia			−0.043	−0.179***	−0.077	0.023		0.201***
			(0.048)	(0.025)	(0.054)	(0.023)		(0.075)
Turkey				−0.840***		−0.608***		−1.032***
				(0.030)		(0.060)		(0.076)
Ukraine		−0.519***						−0.337***
		(0.037)						(0.060)
Kosovo					−0.073**	−0.278***	0.451***	−0.393***
					(0.029)	(0.012)	(0.137)	(0.084)
*Observations*	6,514	13,962	49,935	102,828	27,893	61,591	8,187	18,158
*Pseudo R-Squared*	0.190	0.0793	0.178	0.0760	0.176	0.0798	0.185	0.0856

(1) the dependent variable is “Depression”, (2) the dependent variable is “Life Satisfaction” by considering the sample of people interviewed during the Summer. (3) the dependent variable is “Depression”, (4) the dependent variable is “Life Satisfaction” by considering the sample of people interviewed during the Autumn. (5) the dependent variable is “Depression”, (6) the dependent variable is “Life Satisfaction” by considering the sample of people interviewed during the Winter. (7) the dependent variable is “Depression”, (8) the dependent variable is “Life Satisfaction” by considering the sample of people interviewed during the Spring. Sample survival indicates the marginal effects of the covariates on the survival across waves. Standard errors clustered at country level in parentheses.

*** $$p<0.01$$, ** $$p<0.05$$, * $$p<0.1$$

Country coefficients are those of the corresponding Table 8. Albania is the omitted benchmark. Note that: (1) the dependent variable is “Depression”, (2) the dependent variable is “Life Satisfaction” by considering the sample of people interviewed during the Summer. (3) the dependent variable is “Depression”, (4) the dependent variable is “Life Satisfaction’ by considering the sample of people interviewed during the Autumn. (5) the dependent variable is “Depression”, (6) the dependent variable is “Life Satisfaction” by considering the sample of people interviewed during the Winter. (7) the dependent variable is “Depression”, (8) the dependent variable is “Life Satisfaction” by considering the sample of people interviewed during the Spring. Sample survival indicates the marginal effects of the covariates on the survival across waves. Standard errors clustered at country level in parentheses

## Footnotes

1 ISCED is the International Standard Classification of Education created by UNESCO to harmonize education levels of different countries into common categories (those corresponding to the education dummies introduced in our estimate). For details see http://uis.unesco.org/en/topic/international-standard-classification-education-isced.

2 A panel with repeated observations over time for the same individual would have been better to test adaptation. Unfortunately we do not have this information in our dataset. What we have is self-declared adaptation based on the following “resilience” question. “If things go wrong, time to get back to normal” and the possible answers are “very long time, long time, neither long nor short, short time, very short time”

3 As is well known depression does not only impact on subjective wellbeing as it also has severe social and economic costs for the society. Sobocki et al. (2006) find that direct and indirect health costs of depression (without considering effects on labor productivity) amount to around 42 billion euros corresponding to a cost of 253 euros per inhabitant and to 1% of European GDP in the 28 European countries considered in their research. Evans-Lacko and Knapp (2016) investigate the impact of depression in the workplace in terms of absenteeism and pre-senteeism and find that they have an impact in the range between 0.1 and 4.9 percent of country GDP according to different countries. Stewart et al. (2003) estimate the impact of depression on labor costs in the US workforce and find that workers with depression reported significantly more total health-related lost productive time than those without depression. In addition to it, the US workers with depression employed in the previous week costed employers an estimated $44 billion per year in lost productive time, an excess of $31 billion per year compared with peers without depression.

## References

[CR1] Álvarez B, Miles-Touya D (2016). Time allocation and women’s life satisfaction: Evidence from Spain. Social Indicators Research.

[CR2] American Psychological Association. (2014). The road to resilience. Washington, DC: American Psychological Association; Retrieved from http://www.apa.org/helpcenter/road-resilience.aspx.

[CR21] Archer T, Adrianson L, Plancak A, Karlsson E (2007). Influence of affective personality on cognition-mediated emotional processing: Need for empowerment. The European Journal of Psychiatry.

[CR3] Balcar J (2014). Soft skills and their wage returns: Overview of empirical literature. Review of Economic Perspectives.

[CR4] Blanchflower David G, Oswald Andrew J (2008). Is well-being U-shaped over the life cycle?. Social Science & Medicine.

[CR5] Başlevent C, Kirmanoğlu H (2017). Gender inequality in Europe and the life satisfaction of working and non-working women. Journal of Happiness Studies.

[CR6] Brody Leslie R, Hall Judith A (2008). Gender and emotion in context. Handbook of Emotions.

[CR7] Bryant Fred B, Yarnold Paul R, Grimm Laurence G (1996). Toward a measurement model of the affect intensity measure: A three-factor structure. Journal of Research in Personality.

[CR8] Chen H, Pine DS, Ernst M, Gorodetsky E, Kasen S, Gordon K, Goldman D, Cohen P (2013). The MAOA gene predicts happiness in women. Progress in Neuro-Psychopharmacology & Biological Psychiatry.

[CR9] Carstensen LL, Shavit YZ, Barnes JT (2020). Age advantages in emotional experience persist even under threat from the COVID-19 pandemic. Psychological Science.

[CR10] Clark A, Diener E, Georgellis Y, Lucas R (2008). Lags and Leads in Life Satisfaction: A Test of the Baseline Hypothesis. Economic Journal.

[CR11] Chodorow Nancy (1978). Mothering, object-relations, and the female oedipal configuration. Feminist Studies.

[CR12] Croson Rachel, Gneezy Uri (2009). Gender differences in preferences. Journal of Economic Literature.

[CR13] Diener Ed, Sandvik Ed, Larsen Randy J (1985). Age and sex effects for emotional intensity. Developmental Psychology.

[CR14] Evans-Lacko, S., & Knapp, M. (2016). Cost of depression in the workplace across eight diverse countries –collectively US\$250 billion. LSE Health and Social Care (15 Nov 2016). Website.

[CR15] Frijters Paul, Johnston David W, Shields Michael A (2011). Life satisfaction dynamics with quarterly life event data. Scandinavian Journal of Economics.

[CR16] Frijters Paul, Beatton Tony (2012). The mystery of the U-shaped relationship between happiness and age. Journal of Economic Behavior & Organization.

[CR17] Fujita Frank, Diener Ed, Sandvik E (1991). Gender differences in negative affect and well-being: the case for emotional intensity. Journal of Personality and Social Psychology.

[CR18] Gilligan, C (1982). In a different voice: Psychological theory and women’s development. 6349031

[CR19] Inglehart R (2002). Gender, Aging, and Subjective Well-Being. International Journal of Comparative Sociology.

[CR20] Keller, E. F., & Scharff-Goldhaber, G. (1987). Reflections on gender and science. 10.1119/1.15186.

[CR22] Kessler RC, McGonagle KA, Swartz M (1993). Sex and depression in the National Comorbidity Survey. 1: Lifetime prevalence, chronicity and recurrence. Journal of Affective Disorders.

[CR23] Lorant V, Croux C, Weich S, Deliège D, Mackenbach J, Ansseau M (2007). Depression and socio-economic risk factors: 7-year longitudinal population study. British Journal of Psychiatry.

[CR24] Matteucci, N., & Lima, S. V. (2016). Women and happiness. In *Handbook of Research Methods and Applications in Happiness and Quality of Life*. Edward Elgar Publishing. 10.4337/9781783471171.00025.

[CR25] Merchant, C. (1979). The Death of Nature Women, Ecology, and the Scientific Revolution: A Feminist Reappraisal of the Scientific Revolution.

[CR26] Rudolf Noble E (2005). Depression in women. Metabolism.

[CR27] Nolen-Hoeksema S, Rusting CL, Kahneman D, Diener E, Schwarz N (1999). Gender Differences in Well-Being. Well-Being: The Foundations of Hedonic Psychology.

[CR28] Nordenmark M (2018). The importance of job and family satisfaction for happiness among women and men in different gender regimes. Societies.

[CR29] Sobocki P, Jönsson B, Angst J, Rehnberg C (2006). Cost of Depression in Europe. Journal of Mental Health Policy and Economics.

[CR30] Stiglitz Joseph E, Weiss Andrew (1981). Credit rationing in markets with imperfect information. The American Economic Review.

[CR31] Stewart WF, Ricci JA, Chee E, Hahn SR, Morganstein D (2003). Cost of lost productive work time among US workers with depression. JAMA.

[CR32] Weissman MM, Bland RC, Canino GJ (1996). Cross-national epidemiology of major depression and bipolar disorder. JAMA.

